# Disentangling *Trypanosoma cruzi* transmission cycle dynamics through the identification of blood meal sources of natural populations of *Triatoma dimidiata* in Yucatán, Mexico

**DOI:** 10.1186/s13071-019-3819-7

**Published:** 2019-11-29

**Authors:** Joel Israel Moo-Millan, Audrey Arnal, Silvia Pérez-Carrillo, Anette Hernandez-Andrade, María-Jesús Ramírez-Sierra, Miguel Rosado-Vallado, Eric Dumonteil, Etienne Waleckx

**Affiliations:** 1 Laboratorio de Parasitología, Centro de Investigaciones Regionales “Dr Hideyo Noguchi”, Universidad Autónoma de Yucatán, Mérida, Mexico; 2 Departamento de Ecología de la Biodiversidad, Instituto de Ecología, Universidad Nacional Autónoma de México, Mexico City, México; 3 Department of Tropical Medicine, Tulane University, School of Public Health and Tropical Medicine, New Orleans, LA USA; 4 Institut de Recherche pour le Développement, UMR INTERTRYP IRD, CIRAD, Université de Montpellier, Montpellier, France

**Keywords:** *Triatoma dimidiata*, Blood feeding sources, *Trypanosoma cruzi*, Transmission cycles, Yucatán, Ecohealth, One Health, Chagas disease, Natural populations

## Abstract

**Background:**

In the Yucatán Peninsula, Mexico, *Triatoma dimidiata* is the main vector of *Trypanosoma cruzi*, the causative agent of Chagas disease. Little effort has been made to identify blood meal sources of *T. dimidiata* in natural conditions in this region, although this provides key information to disentangle *T. cruzi* transmission cycles and dynamics and guide the development of more effective control strategies. We identified the blood meals of a large sample of *T. dimidiata* bugs collected in different ecotopes simultaneously with the assessment of bug infection with *T. cruzi*, to disentangle the dynamics of *T. cruzi* transmission in the region.

**Methods:**

A sample of 248 *T. dimidiata* bugs collected in three rural villages and in the sylvatic habitat surrounding these villages was used. DNA from each bug midgut was extracted and bug infection with *T. cruzi* was assessed by PCR. For blood meal identification, we used a molecular assay based on cloning and sequencing following PCR amplification with vertebrate universal primers, and allowing the detection of multiple blood meals in a single bug.

**Results:**

Overall, 28.7% of the bugs were infected with *T. cruzi*, with no statistical difference between bugs from the villages or from sylvatic ecotopes. Sixteen vertebrate species including domestic, synanthropic and sylvatic animals, were identified as blood meal sources for *T. dimidiata*. Human, dog and cow were the three main species identified, in bugs collected in the villages as well as in sylvatic ecotopes. Importantly, dog was highlighted as the main blood meal source after human. Dog was also the most frequently identified animal together with human within single bugs, and tended to be associated with the infection of the bugs.

**Conclusions:**

Dog, human and cow were identified as the main mammals involved in the connection of sylvatic and domestic transmission cycles in the Yucatán Peninsula, Mexico. Dog appeared as the most important animal in the transmission pathway of *T. cruzi* to humans, but other domestic and synanthropic animals, which most were previously reported as important hosts of *T. cruzi* in the region, were evidenced and should be taken into account as part of integrated control strategies aimed at disrupting parasite transmission.

## Background

Chagas disease, also known as American trypanosomiasis, is a life-threatening zoonosis caused by the parasite *Trypanosoma cruzi*. It is one of the main neglected tropical diseases (NTDs) and a major public health problem in the Americas, where six to seven million people are estimated to be infected with the parasite [1]. *Trypanosoma cruzi* is transmitted to more than 180 mammal species (including humans) by blood-sucking bugs called triatomines, from which 31 species are currently reported in Mexico [2, 3]. In this country, active transmission is reported in most of the territory and the most recent estimates suggest a national seroprevalence of 3.38%, suggesting that around four million people carry the parasite in the country, and highlighting the urgency of establishing Chagas disease surveillance and control as a key national public health priority in Mexico [4].

In the Yucatán Peninsula, the main vector of *T. cruzi* is *Triatoma dimidiata*, which is one of the most widespread Triatominae taxa with a native range extending from Colombia to southern Mexico [5]. While *T. dimidiata* may colonize houses in some regions, it also presents an intrusive behavior in multiple regions and most particularly in Yucatán. In this region, located in southern Mexico, it lives mainly in sylvatic ecotopes and to a lesser extent in peridomestic habitats, but frequently enters inside homes, on a seasonal basis, without establishing large colonies [6–8]. In this context, vector control based on massive insecticide spraying has limited efficacy and is not sustainable [9, 10] while alternative strategies, based for example on Ecohealth/One Health approaches are giving promising results [11, 12].

Curiously, little effort has been made to identify blood meal sources of *T. dimidiata* in natural conditions in this region, although this provides key information to disentangle *T. cruzi* transmission cycles and dynamics, evaluate the risk of human infection, and guide the development of more effective control strategies. In prior studies performed in Yucatán, generalist feeding habits have been reported for this species, including mammals, birds and reptiles uncovering domestic, synanthropic and sylvactic animals in its diet, but available information is still limited, mainly because of sample sizes and/or sample composition and/or techniques that have been used [13–16]. Here, simultaneously with the assessment of the infection of the triatomine bugs with *T. cruzi*, we identified the blood meal sources in a large sample of *T. dimidiata* collected in different ecotopes using a molecular assay allowing for the identification of multiple blood meals in a single bug [17–20] to better understand the dynamics of *T. cruzi* transmission to humans in the region.

## Methods

### *Triatoma dimidiata* specimens

A sample composed of 248 *T. dimidiata* specimens including 237 adults (114 males and 123 females), and 11 nymphs (all fifth-instar nymphs, N5) was used. These were collected in the field during entomological surveillance following a pilot vector control intervention during years 2013–2015 [11] in the rural villages of Bokobá (21°00ʹ27″N, 89°10ʹ47″W), Teya (21°02ʹ55″N, 89°04ʹ25″W) and Sudzal (20°52ʹ19″N, 88°59ʹ20″W), as well as using white light traps [21] in the sylvatic habitat surrounding these villages (up to 8 km from the villages), and composed of low and medium subdeciduous tropical forest (locally known as “monte”). The regional climate in the area is warm and humid, with an average annual temperature of 26 °C and 1150 mm of rainfall [22]. Additionally, three *T. dimidiata* reared in the laboratory and fed on pigeons/doves were used as controls of the whole process described below, from extraction of the DNA contained in bug midguts to blood meal identification. These include one male and two N5. Detailed information of each *T. dimidiata* specimen is provided in Additional file 1: Table S1.

### DNA extraction and quantification

DNA from each bug midgut was isolated by using the DNeasy Blood and Tissue kit (Qiagen, Valencia, CA, USA), following manufacturer instructions. Purified DNA was quantified using a BioSpec-nano Spectrophotometer (Shimadzu Biotech, Kyoto, Japan).

### Infection of *T. dimidiata* with *T. cruzi*

*Trypanosoma cruzi* infection status of the bugs was assessed by amplifying parasite DNA from each bug midgut by PCR using TCZ primers [23]. Amplifications were performed in a T100^TM^ thermocycler (Biorad, Hercules, CA, USA) in a volume of 20 µl containing 30 ng of DNA, 0.2 µM of each primer, and 5 µl of 2× DreamTaq Green PCR Master Mix (Thermo Fisher Scientific, Waltham, MA, USA). The cycling parameters were an initial denaturation step at 94 °C for 10 min; 30 cycles at 94 °C (20 s), 57 °C (10 s), and 72 °C (30 s); and a 7 min final extension at 72 °C. Positive (purified *T. cruzi* DNA) and negative (ultrapure H_2_O) controls were included for each PCR. After amplification, PCR products were separated by electrophoresis on a 1.0% agarose gel containing ethidium bromide and visualized by UV transillumination. The PCR was considered invalid if the controls did not give the expected results. The presence or absence of the specific 188-bp fragment indicated the positive or negative (in absence of PCR inhibition, see below) infection status of the bug. Additionally, the DNA of each negative bug was tested for PCR inhibition. For this, another PCR was performed as described above but adding 1 µl of positive control to the reaction mixture. If the expected 188-bp fragment was not visualized after electrophoresis of the PCR product, the sample was classified as “inhibited”.

### Blood meal detection and identification

Blood meals were detected by amplifying the DNA extracted from each bug midgut by PCR using L1085 and H1259 primers as previously described [17–20]. These primers are universal for vertebrate mitochondrial DNA and are designed for the amplification of a ~ 215-bp fragment of the *12S* ribosomal RNA gene [24]. PCR amplifications were performed as above but with ~ 60 ng of DNA and 0.35 µM of each primer in the reaction. The cycling parameters were an initial denaturation step at 95 °C for 5 min; followed by 35 cycles at 95 °C (30 s), 60 °C (15 s), and 72 °C (30 s); and a 10 min final extension at 72 °C. Positive (human DNA) and negative (ultrapure H_2_O) controls were always included. After amplification, PCR products were electrophoresed as described above and the PCR was repeated if the controls did not give the expected results. The visualization of a ~ 215-bp fragment indicated the presence of at least one vertebrate blood meal in the corresponding bug midgut. In this case, the corresponding bug was considered as fed and the PCR product was purified using Wizard® SV Gel and PCR Clean-Up System (Promega, Madison, WI, USA), quantified as above, and cloned to enable the detection of potential multiple blood sources in a single bug. For cloning, the p-GEM®-T Vector System (Promega) was used following the manufacturer’s instructions, using a DNA vector ratio of 3:1 in the ligation step. Background cloning controls were included as indicated by the manufacturer and the cloning was repeated if the controls did not give the expected results. After transformation, we randomly selected, when available, up to eight transformants (white bacterial colonies) per individual bug. DNA extraction of the transformants was then performed by resuspending each selected colony in 25 µl of ultrapure H_2_O and heating at 95 °C for 5 min. After DNA extraction from the transformants, the 12S cloned fragments were re-amplified using the same primers and conditions described above and PCR products from each clone were purified as described above. The direct sequencing of both strands of PCR products was then performed (Eton Bioscience Inc., San Diego, CA, USA). Sequences of both strands were aligned using Clustal-W [25] provided in BioEdit version 7.2.5 [26], and corrected in case of any discrepancy by analyzing the corresponding chromatograms. Blood meal sources were inferred by using BLAST with ≥ 95% identity as the criterion for a match. In each case, the species corresponding to the highest identity was reported as the blood meal source. Nevertheless, in case of ambiguity between different species, only the identification to the genus level was reported. This occurred when (i) a sequence presented exactly the same highest identity with different species (belonging to the same genus) present in Yucatán; and (ii) a sequence presented the best identity with a species not reported in Yucatán, but belonging to a genus present in Yucatán. Moreover, when a sequence presented the same highest identity with different species belonging to the same genus, but only one of these was reported in Yucatán, only this species was reported as the blood meal source.

To assess blood meal sources diversity, Shannonʼs diversity index (Hʹ = − Σ(ni/N)·ln(ni/N)) was calculated, with ni representing the number of individuals of species/taxon i, N the total number of individuals, and S the total number of species/taxa [27]. Furthermore, to estimate the richness of vertebrate species used as blood sources by *T. dimidiata* in the different ecotopes, rarefaction curves were elaborated using the software Past v.3.23 and Microsoft Excel.

To assess potential transmission cycles of *T. cruzi* parasites by *T. dimidiata* among identified blood source species, a feeding and parasite transmission network was constructed using Cytoscape 3.5. It allowed visualizing the frequency of the identified feeding sources as well as possible pathways for parasite transmission among species when multiple blood meals were detected, since feeding sources can be used as evidence of vector-host contact. Nodes of the network represent the various species identified as feeding sources, and edges link blood meal sources that were found in the same individual midgut content.

### Statistical analyzes

All statistical analyzes were performed in JMP Pro 10 software (SAS Institute Inc., Cary, NC, 2013). Associations of bug development stage, sex, ecotope and locality with *T. cruzi* infection or blood sources were tested using Chi-square tests or Fisher’s exact tests, considering that test results were significant when *P* < 0.05. Blood meal diversity indices were compared using a Student’s t-test as previously described, considering that test result was significant if *P* < 0.05 [28].

## Results

### Infection of *T. dimidiata* with *T. cruzi*

Of the 248 *T. dimidiata* bugs included in this study, 64 were positive for *T. cruzi* infection and 159 were negative, giving an overall prevalence of 28.7% (64/223). The infection status of the remaining 25 bugs could not be determined because of PCR inhibition (25 bugs) (Additional file 1: Table S1). The infection prevalence was 17.9% (10/56) in Bokobá, 37.8% (32/87) in Sudzal and 27.5% (22/80) in Teya. Prevalence in sylvatic bugs was 30.9% (21/68) and prevalence in bugs collected within the villages was 27.7% (43/155). The difference was not significant (*χ*^2^ = 0.228, *df* = 1, *P* = 0.633). Additionally, there were no significant differences in infection prevalence between adult and N5 bugs [28.2% (60/213) and 40.0% (4/10), respectively] and between sexes [males, 26.9% (28/104); females, 29.4% (32/109)] (*χ*^2^ = 0.653 and 0.156 respectively, *df* = 1 in both cases, *P* = 0.419 and 0.693, respectively).

### Nutritional status and identified blood meal sources of *T. dimidiata*

**Fig. 1:**
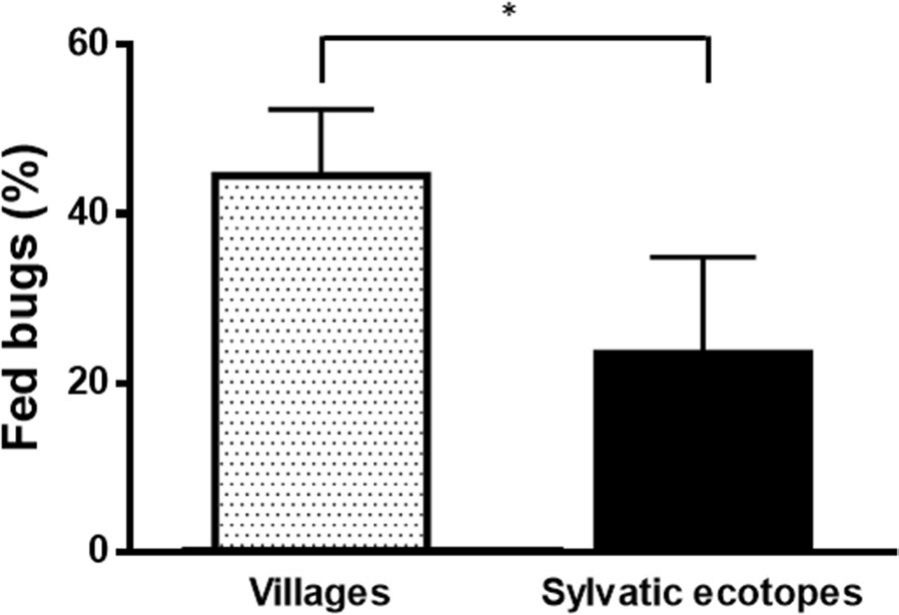
Nutritional status of *T. dimidiata* collected in three rural villages of the Yucatán Peninsula, Mexico, and in the sylvatic ecotopes surrounding these villages. Error bars represent the upper limit of the 95% confidence interval, according to Newcombe, without correction of continuity [29]. The asterisk indicates a significant difference between groups (*χ*^2^ = 8.825, *df* = 1, *P* = 0.003)

Of the 223 bugs which did not show PCR inhibition, 85 (38.1%) were considered as fed because a *12S* amplicon was obtained. A greater proportion of bugs collected in the villages were fed compared to bugs from sylvatic ecotopes [villages: 44.5% (69/155); sylvatic: 23.5% (16/68); *χ*^2^ = 8.825, *df* = 1, *P* = 0.003] (Fig. 1, Table 1).

**Table 1:** Nutritional status and blood meal sources of *T. dimidiata* collected in three rural villages of the Yucatán Peninsula, Mexico, and in the sylvatic ecotopes surrounding these villages

	Villages No. observed/Total no. of bugs (%)	Sylvatic ecotopes No. observed/Total no. of bugs (%)	Total No. observed/Total no. of bugs (%)
PCR-inhibited bugs	18/173 (10.4)	7/75 (9.3)	25/248 (10.1)
Unfed bugs	86/155 (55.5)	52/68 (76.5)	138/223 (61.9)
Fed bugs	69/155 (44.5)	16/68 (23.5)	85/223 (38.1)
Fed bugs with identified blood meal sources	54/69 (78.3)	13/16 (81.3)	67/85 (78.8)
Blood meal sources			
Human (*Homo sapiens*)	28/54 (51.9)	5/13 (38.5)	33/67 (49.3)
Dog (*Canis lupus*)	13/54 (24.1)	2/13 (15.4)	15/67 (22.4)
Cow (*Bos* sp.)	6/54 (11.1)	3/13 (23.1)	9/67 (13.4)
Dove (*Zenaida/Columba* sp.)	3/54 (5.6)	2/13 (15.4)	5/67 (7.5)
Pig (*Sus scrofa*)	4/54 (7.4)	nd	4/67 (6.0)
Mouse (*Mus musculus*)	4/54 (7.4)	nd	4/67 (6.0)
Cat (*Felis catus*)	3/54 (5.6)	nd	3/67 (4.5)
Turkey (*Meleagris gallopavo*)	2/54 (3.7)	nd	2/67 (3.0)
Chicken (*Gallus gallus*)	1/54 (1.9)	nd	1/67 (1.5)
Bat (*Artibeus lituratus*)	1/54 (1.9)	nd	1/67 (1.5)
Peccary (*Pecari tajacu*)	1/54 (1.9)	nd	1/67 (1.5)
Greater grison (*Galictis vittata*)	1/54 (1.9)	nd	1/67 (1.5)
Squirrel (*Sciurus* sp.)	nd	1/13 (7.7)	1/67 (1.5)
Frog (*Rana* sp.)	nd	1/13 (7.7)	1/67 (1.5)
Porcupine (*Coendou* sp.)	nd	1/13 (7.7)	1/67 (1.5)
Deer (*Odocoileus virginianus*)	nd	1/13 (7.7)	1/67 (1.5)

*Notes*: The sums of the percentages of the different blood meal sources are greater than 100 because of multiple blood meal sources detected in various bugs. There was no significant difference in blood meal sources between bugs collected within villages and bugs collected within sylvatic ecotopes (Fisher’s exact test, *P* > 0.05)

*Abbreviations*: nd, not detected

We were able to identify at least one blood meal source in 67 bugs, i.e. approximately in 80% of fed bugs: bugs collected within the villages (78.3%, 54/69); bugs collected within sylvatic ecotopes (81.3%, 13/16) (Table 1). Overall, 16 vertebrate species were identified as blood meal sources for *T. dimidiata*. Up to three different blood meal species per bug were identified, and multiple blood meal species were found in 15/67 bugs [22.4%, with a similar ratio for bugs collected within the villages and those collected within sylvatic ecotopes, 22.2% (12/54) and 23.1% (3/13), respectively]. Six blood meal species were identified from bugs collected in the locality of Bokobá, 14 from bugs collected in Sudzal, and five from bugs collected in Teya. For bugs collected within the villages, 12 blood meal species were identified while 8 blood meal species were identified from bugs collected within sylvatic ecotopes (Table 1). The average number of blood meal sources per bug and the diversity of blood meal sources identified (Shannonʼs diversity index Hʹ) were similar between bugs collected in the villages and in sylvatic ecotopes (average number of blood meal sources per bug: 1.24 *vs* 1.25; Hʹ: 1.87 *vs* 1.89, respectively; *t* = 0.17, *df* = 18, *P* > 0.05).

Among the 12 blood meal sources identified from bugs collected in the villages, human, dog and cow were the three most important (Table 1). Blood of at least one of these vertebrates was found in 77.8% (42/54) of the bugs collected in the villages (Table 1, Additional file 1: Table S1). In this group of bugs, we also identified a variety of other domestic animals (cat, pig and poultry), synanthropic animals (mouse, dove and bat) and sylvatic animals (grison and peccary). There was no significant difference in feeding sources between bugs collected inside and outside the households (Fisher’s exact test, *P* > 0.05). As for bugs collected in the villages, bugs collected in sylvatic ecotopes were mainly fed on humans, dogs and cows, and blood from at least one of these vertebrates was found in 69.2% (9/13) of the bugs (Table 1, Additional file 1: Table S1). In this group of bugs, all other identified blood meal sources corresponded to sylvatic (squirrel, frog, porcupine and deer) or synanthropic (dove) animals. Overall, there was no significant difference in feeding sources between bugs collected within the villages and bugs collected within sylvatic ecotopes (Fisher’s exact test, *P* > 0.05). Detailed information of blood meal sources identified in the bugs collected in each village and ecotope is provided in Additional file 1: Table S1. All the curated sequences obtained in this study for blood meal source identification are provided in Additional file 2.

### Nutritional status and blood meal sources of *T. cruzi*-infected and uninfected *T. dimidiata*

Table 2 provides the number of infected and non-infected insects that were fed and their identified blood meal sources. No statistical difference was found between the nutritional status of *T. cruzi*-infected and non-infected bugs [infected: 39.1% (25/64); uninfected: 37.7% (60/159); *χ*^2^ = 0.034, *df* = 1, *P* = 0.854]. Interestingly, almost one third of the bugs fed on humans were infected with *T. cruzi* (27.3%, 9/22). When looking for an association between *T. cruzi* infection and blood meal sources, we found that it almost reached significance (Fisher’s exact test, *P* = 0.051), while when removing blood meals taken on incompetent hosts (frog, dove, chicken and turkey), it reached significance (Fisher’s exact test, *P* = 0.019), suggesting that some host species may be particularly important for triatomine infection. For example, triatomine infection tended to be associated with feeding on dogs, but our sample size did not allow for a detailed analysis of all host species.

**Table 2:** Nutritional status and blood meal sources of *T. cruzi*-infected and uninfected *T*. *dimidiata*

	Infected bugs No. observed/Total no. of bugs (%)	Uninfected bugs No. observed/Total no. of bugs (%)	Total No. observed/Total no. of bugs (%)
Unfed bugs	39/64 (60.9)	99/159 (62.3)	138/223 (61.9)
Fed bugs	25/64 (39.1)	60/159 (37.7)	85/223 (38.1)
Fed bugs with identified blood meal sources	22/25 (88.0)	45/60 (75.0)	67/85 (78.8)
Blood meal sources			
Human (*Homo sapiens*)	9/22 (40.9)	24/45 (53.3)	33/67 (49.3)
Dog (*Canis lupus*)	9/22 (40.9)	6/45 (13.3)	15/67 (22.4)
Cow (*Bos* sp.)	1/22 (4.5)	8/45 (17.)	9/67 (13.4)
Dove (*Zenaida/Columba* sp.)	1/22 (4.5)	4/45 (8.9)	5/67 (7.5)
Pig (*Sus scrofa*)	nd	4/45 (8.9)	4/67 (6.0)
Mouse (*Mus musculus*)	1/22 (4.5)	3/45 (6.7)	4/67 (6.0)
Cat (*Felis catus*)	1/22 (4.5)	2/45 (4.4)	3/67 (4.5)
Turkey (*Meleagris gallopavo*)	nd	2/45 (4.4)	2/67 (3.0)
Chicken (*Gallus gallus*)	nd	1/45 (2.2)	1/67 (1.5)
Bat (*Artibeus lituratus*)	1/22 (4.5)	nd	1/67 (1.5)
Peccary (*Pecarí tajacu*)	1/22 (4.5)	nd	1/67 (1.5)
Greater grison (*Galictis vittata*)	1/22 (4.5)	nd	1/67 (1.5)
Squirrel (*Sciurus* sp.)	nd	1/45 (2.2)	1/67 (1.5)
Frog (*Rana* sp.)	nd	1/45 (2.2)	1/67 (1.5)
Porcupine (*Coendou* sp.)	1/22 (4.5)	nd	1/67 (1.5)
Deer (*Odocoileus virginianus*)	1/22 (4.5)	nd	1/67 (1.5)

*Notes*: The sums of the percentages of the different blood meal sources are greater than 100 because of multiple blood meal sources detected in various bugs. There was no significant difference in blood meal sources between infected and uninfected bugs (Fisher’s exact test, *P* = 0.051). There was no significant difference in nutritional status between infected and uninfected bugs (*χ*^2^ = 0.034, *df* = 1, *P* = 0.854)

*Abbreviations*: nd, not detected

### Blood sources-*T. dimidiata*–*T. cruzi*-ecotope interactions

**Fig. 2:**
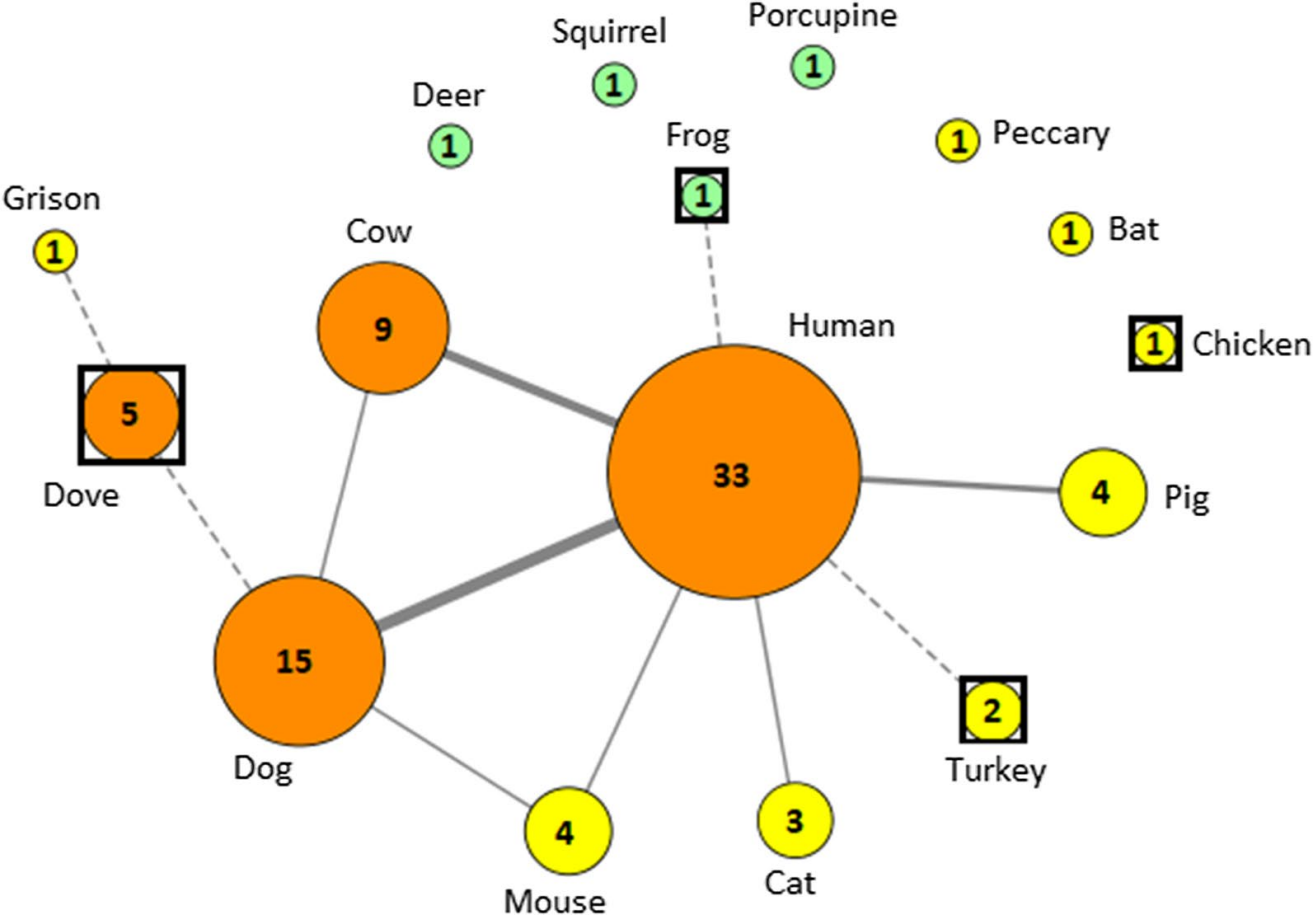
Blood meal sources of *T. dimidiata* and possible *T. cruzi* transmission network. Nodes correspond to blood meal species identified in bugs collected in the villages (yellow symbols), sylvatic ecotopes (green symbols) or in both environments (orange symbols). Species which cannot carry the parasite (birds and amphibians) are represented by circle-in-square shaped nodes, while mammals are represented by circles. Feeding frequency on each species is indicated by the size of the colored area of each corresponding node, and the exact number of times each species was identified is also indicated within each node. Edges link species which were found together within individual bugs, and the width of the lines is proportional to the frequency of the association between species. Solid dark gray lines link mammalian species, among which *T. cruzi* may circulate; dotted black lines involve bird or amphibian species, which only serve as blood sources for the bugs

To further analyze blood sources-*T. dimidiata*–*T. cruzi*-ecotope interactions and assess potential transmission cycles of *T. cruzi* parasites by *T. dimidiata* among identified blood source species, a feeding and parasite transmission network was constructed (Fig. 2). Nodes of the network represent the species identified as blood meal sources, while feeding frequency on each species is indicated by the size of the corresponding node. Edges link species which are found together in multiple blood meals within individual bugs. Since birds and amphibians cannot carry *T. cruzi* parasites, they only play a role as blood meal source for triatomines, which is indicated by dotted edge connections between hosts, while the solid lines between mammals indicate potential parasite transmission pathways. The network shows that the four main blood meal sources (human, dog, cow and dove) were also the only species identified in bugs collected in the villages as well as in bugs collected in sylvatic ecotopes. Moreover, it highlights dogs as the main blood meal source after humans and as the most frequently identified animal together with human within single bugs.

## Discussion

The current international objective concerning Chagas disease, set within the WHO roadmap and the London Declaration on NTDs, is to interrupt vectorial intra-domiciliary transmission in the Americas by 2020 [30, 31]. It is now clear that achievement of this goal will be postponed, particularly because of the role played by intrusive triatomine species on intra-domiciliary transmission, and that innovative control strategies, based on a comprehensive understanding of the ecology of the vectors and of local *T. cruzi* transmission cycles and their dynamics, are needed to achieve such an ambitious target [10, 32, 33].

Here, we identified the blood meal sources of *T. dimidiata* collected in three rural villages of the Yucatán Peninsula and in sylvatic ecotopes surrounding these villages, simultaneously with the assessment of the infection of the bugs with *T. cruzi*, in an effort to disentangle local transmission cycles and their dynamics. The molecular assay used allowed the identification of 16 local vertebrate species involved in *T. cruzi* transmission cycles and/or the maintenance of triatomine populations. Interestingly, we identified four vertebrates which are reported for the first time as blood feeding sources of *T. dimidiata* [13–16, 19], i.e. *Pecari tajacu* (peccary), *Galictis vittata* (greater grison), *Odocoileus virginianus* (deer), and *Rana* sp. (frog). Additionally, the assay used allowed the detection of multiple blood meal sources in various bugs, even if the proportion of bugs with multiple blood meals tended to be lower than in other studies using the same assay [19, 20]. It also allowed confirming that *T. dimidiata* feed on a large variety of animals [16]. The latter, combined with the fact that once triatomines acquire infection with *T. cruzi*, they carry the parasite life-long (as illustrated by the infected bug for which the identified blood source was dove, an incompetent host, see Table 2), may explain why we could not find any statistical association between feeding sources and infection with *T. cruzi*, even if we observed that dog as feeding source tended to be associated with the infection of the bugs.

Human, dog and cow were identified as the main blood meal sources from the bugs collected in the villages as well as from the bugs collected in sylvatic ecotopes. Blood of at least one of these vertebrates was found in more than 75% of the bugs, suggesting that these three mammals may play an important role in the connection of sylvatic and domestic transmission cycles. However, there were some differences between villages. For instance, in Sudzal, cow was not identified as a blood source, either from the bugs collected in sylvatic ecotopes, or in the village. On the contrary, from the bugs collected in the sylvatic habitat around Sudzal, which is more conserved than the other sylvatic sites sampled and where more sylvatic species can be observed, cow was “replaced” by a variety of sylvatic animals, which were not identified in the other sylvatic sites. This evidences that the feeding behavior also depends on the local availability of blood sources and the way the sylvatic ambient is managed by villagers.

The presence of human blood in bugs collected within sylvatic ecotopes was not surprising, as human blood has previously been detected in high proportions in sylvatic specimens of other triatomine species such as *T. infestans* in Bolivia [34, 35], or as the main blood meal source in sylvatic *T. dimidiata* in Guatemala [19]. Nevertheless, since there are risks of contamination with human DNA in studies dealing with blood host identification [17], we added laboratory-reared triatomines fed on pigeons, which were processed simultaneously (from DNA extraction to sequencing) with the bugs of our sample, in order to rule out or estimate the level of possible contamination. Six percent of the obtained sequences (one sequence from one clone from one of the three laboratory-reared triatomines) after the whole procedure corresponded to human instead of pigeon/dove (Additional file 1: Table S1), evidencing some low level of contamination in spite of the extreme precaution taken in sample handling, using highest standard clean procedures. The detection of human blood meals in bugs collected in the sylvatic ecotopes could be explained, as previously proposed, by (i) the movement of bugs between the sylvatic environment and the villages; and/or (ii) the intrusion of humans into the sylvatic environment [34, 36, 37]. Here, it is worth noting that in sylvatic bugs, apart from humans, dogs (which can accompany humans in sylvatic ecotopes during activities such as hunting), and cows (which are close to two of the sampled sites and can enter deeply in the sylvatic environment), no other domestic animals were detected, suggesting that the presence of human, dog and cow blood is more likely due to an intrusion of these mammals into the sylvatic environment than to the movement of the bugs between the sylvatic environment and the villages. This does not question the intrusive behavior of *T. dimidiata* in the Yucatán Peninsula which is well described [6–8, 36–38], but suggests that the vectors invading the villages do not come back as deep into the sylvatic environment as the sylvatic sites sampled in the present study, or do it in a range of time larger than our capacity to detect old blood meal sources. In agreement with previous estimates, *T. dimidiata* takes one (at least partial) blood meal every 3–7 days in natural conditions [16]. Here, we detected up to three blood meals within a single bug. We can thus estimate that feeding patterns identified through our assay reflects the feeding behavior of each bug during the last three to 21 days before being collected. Interestingly, sylvatic blood meal sources (peccary and greater grison) were identified from bugs collected in the villages, likely reflecting movement of bugs between sylvatic ecotopes and villages. Nevertheless, the intrusion of these sylvatic animals into the villages cannot be discarded.

One of the proposed hypotheses to explain the seasonal intrusion of *T. dimidiata* into human settlements is a reduction in sylvatic blood sources availability [38]. Interestingly, we found that the proportion of fed sylvatic bugs was much lower than that of bugs collected in the villages (Fig. 1), in agreement with a previous report indicating a rather poor nutritional status of sylvatic bugs and a limited improvement in domestic bugs [38]. This somewhat supports this hypothesis, even if the way sylvatic bugs were collected can bias their nutritional status as only starved bugs are expected to fly to light traps [39].

The network built from the identification of blood meal sources and the detection of multiple blood meals in various bugs allowed incriminating dog as the main host in the transmission pathway of *T. cruzi* to humans (Fig. 2), as in other regions [40–46] and also in agreement with the high prevalence of infection of this host, ranging from 9.8 to 34 % in the region [47–49]. Importantly, in the Yucatán Peninsula, dogs have also previously been identified as a risk factor for house infestation by *T. dimidiata* [11, 50]. In our network, cow, mouse, pig and cat were the other vertebrates involved in possible transmission pathways to humans. Apart from cow, for which no data are available concerning *T. cruzi* seroprevalence in the Yucatán Peninsula, all these vertebrates have been reported as important hosts in the region, with variable but generally high *T. cruzi* seroprevalence [51–55]. We thus confirm that all these domestic or synanthropic animals should also be considered as part of integrated control strategies. Taken together, this information strengthens the rationale for controlling *T. cruzi* infection in domestic animals (dogs in particular), to manage their location, or to control synanthropic animals as part of integrated control interventions. Controlling *T. cruzi* infection in domestic animals can be achieved by insecticide treatment [56–59] or vaccination [60, 61], while management of the domestic animals or control of rodents can be part of Ecohealth/One Health approaches [62, 63]. On the other hand, the network indicated that all other species identified played a limited role in parasite transmission to humans, even if this information must be taken with caution and more samples are needed. Interestingly, no bug was found to have been fed on humans together with chicken, while the presence of chickens in peridomestic habitats have also been reported as risk factors for household infestation [11, 50, 64] in the region. This somewhat suggests that chickens could be used in zooprophylactic strategies, as birds are incompetent host of *T. cruzi*. Nevertheless, at this step, this has to be taken with extreme caution as well, as (i) chickens have been shown as a risk factor of finding infected triatomines in households in the region in other studies [64]; (ii) we found in the present study bugs fed on humans and turkeys, another poultry; and (iii) more samples are needed to reinforce the current conclusions. To further assess the positive or negative effects of the management of host community, modeling of the transmission to humans is needed, and data provided by the present study and other dealing with the identification of *T. dimidiata* blood meal sources in the region can be used to feed models including the hosts involved in the transmission to help assessing the effects of different host community managements on *T. cruzi* transmission to humans [65].

Surprisingly, no blood meals were detected from opossums, which have been reported with a high *T. cruzi* infection rate in the region [52, 55], even if infection through other routes such as oral (by feeding triatomines for example) or through anal glands may also play a role for opossums. The absence of detection of opossum in the present study may be due to the limited number of bugs for which blood meals were identified, which may have limited our assessment of feeding source diversity. In this way, the rarefaction curves constructed (Additional file 3: Figure S1) show that we were not able to identify the complete diversity of feeding sources, particularly in the sylvatic habitat. PCR primer bias may also occur, although we successfully amplified control opossum DNA from Yucatán with our primers, and opossum has been previously identified using the same primers [19]. A diminution of opossum populations at the times bugs were collected could also be hypothesized. Or, finally, for some bias we could not detect in the collections, our sample may not contain bugs which live in close association with opossums.

Analysis of additional populations of *T. dimidiata*, and of the seasonal variations in feeding profiles should provide additional information to refine local feeding and transmission networks. Interestingly, we recently performed an innovative study using a metabarcoding approach based on next-generation sequencing to simultaneously identify blood meal sources, *T. cruzi* genetic diversity, *T. dimidiata* genetic subgroups, and microbiome composition of triatomine midguts [16]. This pilot study, performed on a small sample of the same study area, evidenced a very high sensitivity in blood meal source detection (up to seven host species identified within a single bug) and highlighted the same main species as the present study in the hypothetical transmission pathways of *T. cruzi* transmission to humans in the Yucatán Peninsula. This kind of approach, or other based on next-generation sequencing strategies should provide valuable information to refine feeding and transmission networks in the future, and understand their dynamics [16, 66–69].

## Conclusions

In the present study, we confirmed the risk of transmission of *T. cruzi* to humans in the Yucatán Peninsula. Humans were the main feeding source identified in the bugs collected, and almost 30% of the bugs that fed on humans were infected with *T. cruzi*. Dog, human and cow were identified as the main mammals involved in the connection of sylvatic and domestic transmission cycles. Dog appeared as the most important animal in the transmission pathway of *T. cruzi* to humans, but other domestic (cow, cat, pig) and synanthropic (mouse) animals, which have previously been reported as important hosts of *T. cruzi* in the region, were evinced and should be considered as part of integrated control strategies aimed at disrupting parasite transmission.

## Supplementary information

**Additional file 1: Table S1.** Detailed information of *Triatoma dimidiata* specimens included in this study.

**Additional file 2.** Curated sequences obtained in this study for *Triatoma dimidiata* blood meal source identification.

**Additional file 3: Figure S1.** Rarefaction curves of *Triatoma dimidiata* blood meal source diversity.

## Data Availability

All data generated and analyzed during this study are included in this published article and its additional files.

## Abbreviations

DNA: deoxyribonucleic acid
RNA: ribonucleic acid
PCR: polymerase chain reaction
NTDs: neglected tropical diseases
N5: fifth instar nymphs
BLAST: basic local alignment search tool
NumtS: nuclear insertions of mitochondrial origin

## References

[1] WHO. Chagas disease (American trypanosomiasis), Fact sheet No. 340. 2019. http://www.who.int/mediacentre/factsheets/fs340/en/. Accessed 17 July 2019.

[2] Noireau F, Diosque P, Jansen AM (2009). *Trypanosoma cruzi*: adaptation to its vectors and its hosts. Vet Res..

[3] Ramsey JM, Peterson AT, Carmona-Castro O, Moo-Llanes DA, Nakazawa Y, Butrick M (2015). Atlas of Mexican Triatominae (Reduviidae: Hemiptera) and vector transmission of Chagas disease. Mem Inst Oswaldo Cruz..

[4] Arnal A, Waleckx E, Rico-Chávez O, Herrera C, Dumonteil E (2019). Estimating the current burden of Chagas disease in Mexico: a systematic review and meta-analysis of epidemiological surveys from 2006 to 2017. PLoS Negl Trop Dis..

[5] Dorn PL, Monroy C, Curtis A (2007). *Triatoma dimidiata* (Latreille, 1811): a review of its diversity across its geographic range and the relationship among populations. Infect Genet Evol..

[6] Dumonteil E, Gourbiere S, Barrera-Perez M, Rodriguez-Felix E, Ruiz-Pina H, Banos-Lopez O (2002). Geographic distribution of *Triatoma dimidiata* and transmission dynamics of *Trypanosoma cruzi* in the Yucatán Peninsula of Mexico. Am J Trop Med Hyg..

[7] Gourbiere S, Dumonteil E, Rabinovich JE, Minkoue R, Menu F (2008). Demographic and dispersal constraints for domestic infestation by non-domicilated Chagas disease vectors in the Yucatán Peninsula, Mexico. Am J Trop Med Hyg..

[8] Waleckx E, Pasos-Alquicira R, Ramirez-Sierra MJ, Dumonteil E (2016). Sleeping habits affect access to host by Chagas disease vector *Triatoma dimidiata*. Parasites Vectors..

[9] Flores-Ferrer A, Marcou O, Waleckx E, Dumonteil E, Gourbiere S (2018). Evolutionary ecology of Chagas disease; what do we know and what do we need?. Evol Appl..

[10] Waleckx E, Gourbiere S, Dumonteil E (2015). Intrusive versus domiciliated triatomines and the challenge of adapting vector control practices against Chagas disease. Mem Inst Oswaldo Cruz..

[11] Waleckx E, Camara-Mejia J, Jesus Ramirez-Sierra M, Cruz-Chan V, Rosado-Vallado M, Vazquez-Narvaez S (2015). An innovative ecohealth intervention for Chagas disease vector control in Yucatán, Mexico. Trans R Soc Trop Med Hyg..

[12] Waleckx E, Pérez-Carrillo S, Chávez-Lazo S, Pasos-Alquicira R, Cámara-Heredia M, Acuña-Lizama J (2018). Non-randomized controlled trial of the long-term efficacy of an Ecohealth intervention against Chagas disease in Yucatán, Mexico. PLoS Negl Trop Dis..

[13] Guzman-Tapia Y, Ramirez-Sierra MJ, Dumonteil E (2007). Urban infestation by *Triatoma dimidiata* in the city of Mérida, Yucatán, Mexico. Vector Borne Zoonotic Dis..

[14] Guzman-Marin ES, Barrera-Pérez MA, Rodriguez-Felix ME, Zavala-Velazquez JE (1992). Hábitos biológicos de *Triatoma dimidiata* en el estado de Yucatán, México. Rev Biomed..

[15] Quintal RE, Polanco GG (1977). Feeding preferences of *Triatoma dimidiata maculipennis* in Yucatán, Mexico. Am J Trop Med Hyg..

[16] Dumonteil E, Ramirez-Sierra MJ, Perez-Carrillo S, Teh-Poot C, Herrera C, Gourbiere S (2018). Detailed ecological associations of triatomines revealed by metabarcoding and next-generation sequencing: implications for triatomine behavior and *Trypanosoma cruzi* transmission cycles. Sci Rep..

[17] Gorchakov R, Trosclair LP, Wozniak EJ, Feria PT, Garcia MN, Gunter SM (2016). *Trypanosoma cruzi* infection prevalence and bloodmeal analysis in triatomine vectors of Chagas disease from rural peridomestic locations in Texas, 2013–2014. J Med Entomol..

[18] Stevens L, Dorn PL, Hobson J, de la Rua NM, Lucero DE, Klotz JH (2012). Vector blood meals and Chagas disease transmission potential, United States. Emerg Infect Dis..

[19] Stevens L, Monroy MC, Rodas AG, Dorn PL (2014). Hunting, swimming, and worshiping: human cultural practices illuminate the blood meal sources of cave dwelling Chagas vectors (*Triatoma dimidiata*) in Guatemala and Belize. PLoS Negl Trop Dis..

[20] Waleckx E, Suarez J, Richards B, Dorn PL (2014). *Triatoma sanguisuga* blood meals and potential for Chagas disease, Louisiana, USA. Emerg Infect Dis..

[21] Rebollar-Tellez EA, Reyes-Villanueva F, Escobedo-Ortegon J, Balam-Briceno P, May-Concha I (2009). Abundance and nightly activity behavior of a sylvan population of *Triatoma dimidiata* (Hemiptera: Reduviidae: Triatominae) from the Yucatán, Mexico. J Vector Ecol..

[22] Vidal-Zepeda R (1990). Atlas Nacional de México.

[23] Moser DR, Kirchhoff LV, Donelson JE (1989). Detection of *Trypanosoma cruzi* by DNA amplification using the polymerase chain reaction. J Clin Microbiol..

[24] Kitano T, Umetsu K, Tian W, Osawa M (2007). Two universal primer sets for species identification among vertebrates. Int J Legal Med..

[25] Thompson JD, Higgins DG, Gibson TJ (1994). CLUSTAL-W - Improving the sensitivity of progressive multiple sequence alignment through sequence weighting, position-specific gap penalties and weight matrix choice. Nucleic Acids Res..

[26] Hall TA (1999). BioEdit: a user-friendly biological sequence alignment editor and analysis program for Windows 95/98/NT. Nucl Acids Symp Ser..

[27] Shannon CE, Weaver W (1949). The mathematical theory of communication.

[28] Hutcheson K (1970). A test for comparing diversities based on the Shannon formula. J Theor Biol..

[29] Newcombe RG (1998). Two-sided confidence intervals for the single proportion: comparison of seven methods. Stat Med..

[30] WHO. Accelerating work to overcome the global impact of neglected tropical diseases: a roadmap for implementation. Geneva: World Health Organization; 2012. https://www.who.int/neglected_diseases/NTD_RoadMap_2012_Fullversion.pdf. Accessed 18 Sept 2019.

[31] Uniting to Combat Neglected Tropical Diseases. London declaration on neglected tropical diseases. 2012. https://www.who.int/neglected_diseases/London_Declaration_NTDs.pdf. Accessed 18 Sept 2019.

[32] Abad-Franch F (2016). A simple, biologically sound, and potentially useful working classification of Chagas disease vectors. Mem Inst Oswaldo Cruz..

[33] Peterson JK, Yoshioka K, Hashimoto K, Caranci A, Gottdenker N, Monroy C (2019). Chagas disease epidemiology in Central America: an update. Curr Trop Med Rep..

[34] Buitrago R, Bosseno MF, Depickere S, Waleckx E, Salas R, Aliaga C (2016). Blood meal sources of wild and domestic *Triatoma infestans* (Hemiptera: Reduviidae) in Bolivia: connectivity between cycles of transmission of *Trypanosoma cruzi*. Parasites Vectors..

[35] Buitrago R, Bosseno MF, Waleckx E, Brémond P, Vidaurre P, Zoveda F (2013). Risk of transmission of *Trypanosoma cruzi* by wild *Triatoma infestans* (Hemiptera: Reduviidae) in Bolivia supported by the detection of human blood meals. Infect Genet Evol.

[36] Barbu C, Dumonteil E, Gourbiere S (2010). Characterization of the dispersal of non-domiciliated *Triatoma dimidiata* through the selection of spatially explicit models. PLoS Negl Trop Dis..

[37] Dumonteil E, Tripet F, Ramirez-Sierra MJ, Payet V, Lanzaro G, Menu F (2007). Assessment of *Triatoma dimidiata* dispersal in the Yucatán Peninsula of Mexico by morphometry and microsatellite markers. Am J Trop Med Hyg..

[38] Payet V, Ramirez-Sierra MJ, Rabinovich J, Menu F, Dumonteil E (2009). Variations in sex ratio, feeding, and fecundity of *Triatoma dimidiata* (Hemiptera: Reduviidae) among habitats in the Yucatán Peninsula, Mexico. Vector Borne Zoonotic Dis..

[39] Noireau F, Abad-Franch F, Valente SAS, Dias-Lima A, Lopes CM, Cunha V (2002). Trapping Triatominae in sylvatic habitats. Mem Inst Oswaldo Cruz..

[40] Cohen JE, Gurtler RE (2001). Modeling household transmission of American trypanosomiasis. Science..

[41] Crisante G, Rojas A, Teixeira MMG, Añez N (2006). Infected dogs as a risk factor in the transmission of human *Trypanosoma cruzi* infection in western Venezuela. Acta Trop..

[42] Gurtler RE, Cardinal MV (2015). Reservoir host competence and the role of domestic and commensal hosts in the transmission of *Trypanosoma cruzi*. Acta Trop..

[43] Gurtler RE, Cecere MC, Lauricella MA, Cardinal MV, Kitron U, Cohen JE (2007). Domestic dogs and cats as sources of *Trypanosoma cruzi* infection in rural northwestern Argentina. Parasitology..

[44] Pineda V, Saldana A, Monfante I, Santamaria A, Gottdenker NL, Yabsley MJ (2011). Prevalence of trypanosome infections in dogs from Chagas disease endemic regions in Panama, Central America. Vet Parasitol..

[45] Ramirez JD, Turriago B, Tapia-Calle G, Guhl F (2013). Understanding the role of dogs (*Canis lupus familiaris*) in the transmission dynamics of *Trypanosoma cruzi* genotypes in Colombia. Vet Parasitol..

[46] Enriquez GF, Bua J, Orozco MM, Wirth S, Schijman AG, Gurtler RE (2014). High levels of *Trypanosoma cruzi* DNA determined by qPCR and infectiousness to *Triatoma infestans* support dogs and cats are major sources of parasites for domestic transmission. Infect Genet Evol..

[47] Jimenez-Coello M, Poot-Cob M, Ortega-Pacheco A, Guzman-Marin E, Ramos-Ligonio A, Sauri-Arceo CH (2008). American Trypanosomiasis in dogs from an urban and rural area of Yucatán, Mexico. Vector Borne Zoonotic Dis..

[48] Jimenez-Coello M, Guzman-Marin E, Ortega-Pacheco A, Acosta-Viana KY (2010). Serological survey of American trypanosomiasis in dogs and their owners from an urban area of Mérida Yucatán, Mexico. Transbound Emerg Dis..

[49] Cruz-Chan JV, Bolio-Gonzalez M, Colin-Flores R, Ramirez-Sierra MJ, Quijano-Hernandez I, Dumonteil E (2009). Immunopathology of natural infection with *Trypanosoma cruzi* in dogs. Vet Parasitol..

[50] Dumonteil E, Nouvellet P, Rosecrans K, Ramirez-Sierra MJ, Gamboa-Leon R, Cruz-Chan V (2013). Eco-Bio-Social determinants for house infestation by non-domiciliated *Triatoma dimidiata* in the Yucatán Peninsula, Mexico. PLoS Negl Trop Dis..

[51] Panti-May JA, De Andrade RRC, Gurubel-Gonzalez Y, Palomo-Arjona E, Soda-Tamayo L, Meza-Sulu J (2017). A survey of zoonotic pathogens carried by house mouse and black rat populations in Yucatán, Mexico. Epidemiol Infect..

[52] Zavala-Velazquez J, Barrera-Perez M, Rodriguez-Felix ME, Guzman-Marin E, Ruiz-Pina H (1996). Infection by *Trypanosoma cruzi* in mammals in Yucatán, Mexico: a serological and parasitological study. Rev Inst Med Trop Sao Paulo..

[53] Jimenez-Coello M, Acosta-Viana KY, Guzman-Marin E, Ortega-Pacheco A (2012). American Trypanosomiasis infection in fattening pigs from the South-East of Mexico. Zoonoses Public Health..

[54] Jimenez-Coello M, Acosta-Viana KY, Guzman-Marin E, Gomez-Rios A, Ortega-Pacheco A (2012). Epidemiological survey of *Trypanosoma cruzi* infection in domestic owned cats from the tropical southeast of Mexico. Zoonoses Public Health..

[55] Lopez-Cancino SA, Tun-Ku E, De la Cruz-Felix HK, Ibarra-Cerdena CN, Izeta-Alberdi A, Pech-May A (2015). Landscape ecology of *Trypanosoma cruzi* in the southern Yucatán Peninsula. Acta Trop..

[56] Amelotti I, Catala SS, Gorla DE (2012). Effects of fipronil on dogs over *Triatoma infestans*, the main vector of *Trypanosoma cruzi*, causative agent of Chagas disease. Parasitol Res..

[57] Reithinger R, Ceballos L, Stariolo R, Davies CR, Gurtler RE (2006). Extinction of experimental *Triatoma infestans* populations following continuous exposure to dogs wearing deltamethrin-treated collars. Am J Trop Med Hyg..

[58] Reithinger R, Ceballos L, Stariolo R, Davies CR, Gurtler RE (2005). Chagas disease control: deltamethrin-treated collars reduce *Triatoma infestans* feeding success on dogs. Trans R Soc Trop Med Hyg..

[59] Gurtler RE, Ceballos LA, Stariolo R, Kitron U, Reithinger R (2009). Effects of topical application of fipronil spot-on on dogs against the Chagas disease vector *Triatoma infestans*. Trans R Soc Trop Med Hyg..

[60] Quijano-Hernandez IA, Castro-Barcena A, Vazquez-Chagoyan JC, Bolio-Gonzalez ME, Ortega-Lopez J, Dumonteil E (2013). Preventive and therapeutic DNA vaccination partially protect dogs against an infectious challenge with *Trypanosoma cruzi*. Vaccine..

[61] Basombrio MA, Segura MA, Mora MC, Gomez L (1993). Field trial of vaccination against American Trypanosomiasis (Chagas disease) in dogs. Am J Trop Med Hyg..

[62] De Urioste-Stone SM, Pennington PM, Pellecer E, Aguilar TM, Samayoa G, Perdomo HD (2015). Development of a community-based intervention for the control of Chagas disease based on peridomestic animal management: an eco-bio-social perspective. Trans R Soc Trop Med Hyg..

[63] Pellecer MJ, Dorn PL, Bustamante DM, Rodas A, Monroy MC (2013). Vector blood meals are an early indicator of the effectiveness of the Ecohealth approach in halting Chagas transmission in Guatemala. Am J Trop Med Hyg..

[64] Koyoc-Cardena E, Medina-Barreiro A, Escobedo-Ortegon FJ, Rodriguez-Buenfil JC, Barrera-Perez M, Reyes-Novelo E (2015). Chicken coops, *Triatoma dimidiata* infestation and its infection with *Trypanosoma cruzi* in a rural village of Yucatán, Mexico. Rev Inst Med Trop Sao Paulo..

[65] Nouvellet P, Cucunuba ZM, Gourbiere S (2015). Ecology, Evolution and control of Chagas disease: a century of neglected modelling and a promising future. Adv Parasitol..

[66] Orantes LC, Monroy C, Dorn PL, Stevens L, Rizzo DM, Morrissey L (2018). Uncovering vector, parasite, blood meal and microbiome patterns from mixed-DNA specimens of the Chagas disease vector *Triatoma dimidiata*. Plos Negl Trop Dis..

[67] Titcomb GC, Jerde CL, Young HS (2019). High-throughput sequencing for understanding the ecology of emerging infectious diseases at the wildlife-human interface. Front Ecol Evol..

[68] Waleckx E, Arnal A, Dumonteil E, Zamora-Bustillos R, Sandoval-Gío JJ (2019). Metabarcoding, un nuevo enfoque para el estudio de los ciclos de transmisión de la enfermedad de Chagas y la ecología de sus vectores. Contribución de la Biotecnología al Desarrollo de la Península de Yucatán.

[69] Hernandez-Andrade A, Moo-Millan J, Cigarroa-Toledo N, Ramos-Ligonio A, Herrera C, Bucheton B, et al. Metabarcoding, a powerful yet still underestimated approach for the comprehensive study of vector-borne pathogen transmission cycles and their dynamics. In: Claborn D, editor. Current topics in the epidemiology of vector-borne diseases. London: Intech Open; 2020.

