# Supplementary material for: Disentangling Trypanosoma cruzi transmission cycle dynamics through the identification of blood meal sources of natural populations of Triatoma dimidiata in Yucatán, Mexico
Source: Parasit Vectors. 2019 Nov 29;12:572. doi: 10.1186/s13071-019-3819-7 (PMC6884771; doi:10.1186/s13071-019-3819-7)
Supplement: Supplementary file 2 — Additional file 2. Curated sequences obtained in this study for Triatoma dimidiata blood meal source identification. [file 13071_2019_3819_MOESM2_ESM.docx]

**Additional file 2.** **Curated sequences obtained in this study for *Triatoma dimidiata* blood meal source identification**. The name of each sequence indicates the corresponding *T. dimidiata* specimen, clone, and identified blood meal species.

**BOK002**

>Bok002_clone1 (*Zenaida/Columba* sp*.*)

TGGCCCTAAATCTAGATGCTTATACTACTAAAGCATCCGCCTGAGGACTACGAGCACAAACGCTTAAAACTCTAAGGACTTGGCGGTGCCCTAAACCCACCTAGAGGAGCCTGTTCTATAATCGATAACCCACGATACACCCGA

>Bok002_clone2 (*Zenaida/Columba* sp*.*)

AAATCTAGATGCTTATACTACTAAAGCATCCGCCTGAGGGCTACGAGCACAAACGCTTAAAACTCTAAGGACTTGGCGGTGCCCTAAACCCACCTAGAGGAGCCTGTTCTATAATCGATAACCCACGATACACCCGACCACTTCTCG

>Bok002_clone3 (*Zenaida/Columba* sp*.*)

AATCTAGATGCTTATACTACTAAAGCATCCGCCTGAGGACTACGAGCACAAACGCTTAAAACTCTAAGGACTTGGCGGTGCCCTAAACCCACCTAGAGGAGCCTGTTCTATAATCGATAACCCACGATACACCCGAC

>Bok002_clone4 (*Galictis vittata*)

AATAATTCATTCAACAAAATTATCTGCCAGAGAACTACTAGCAACAGCCTAAAACTCAAAGGACTTGGCGGTGCTTTACATCCCTCTAGAGGAGCCTGTTCTATAATCGATAAACCCCGATAAACCTTACC

**BOK011**

>Bok011_clone1 (*Homo sapiens*)

CTCAACAGTTAAATCAACAAAACTGCTCGCCAGAACACTACGAGCCACAGCTTAAAACTCAAAGGACCTGGCGGTGCTTCATATCCCTCTAGAGGAGCCTGTTCTGTAA

>Bok011_clone2 (*Homo sapiens*)

GCTCGCCAGAACACTACGAGCCACAGCTTAAAACTCAAAGGACCTGGCGGTGCTTCATATCCCTCTAGAGGAGCCTGTTCTGTAATCGATAAACCCCG

>Bok011_clone3 (*Homo sapiens*)

GCTCGCCAGAACACTACGAGCCACAGCTTAAAACTCAAAGGACCTGGCGGTGCTTCATATCCCTCTAGAGGAGCCTGTTCTGTAATCGATAAACCCCGA

>Bok011_clone4 (*Homo sapiens*)

ACAAAACTGCTCGCCAGAACACTACGAGCCACAGCTTAAAACTCAAAGGACCTGGCGGTGCTTCATATCCCTCTAGAGGAGCCTGTTCTGTAATCGATAAACCCCGA

>Bok011_clone5 (*Homo sapiens*)

AAACTGCTCGCCAGAACACTACGAGCCACAGCTTAAAACTCAAAGGACCTGGCGGTGCTTCATATCCCTCTAGAGGAGCCTGTTCTGTAATCG

>Bok011_clone6 (*Homo sapiens*)

GCTCGCCAGAACACTACGAGCCACAGCTTAAAACTCAAAGGACCTGGCGGTGCTTCATATCCCTCTAGAGGAGCCTGTTCTGTAATCG

>Bok011_clone7 (*Homo sapiens*)

ACAAAACTGCTCGCCAGAACACTACGAGCCACAGCTTAAAACTCAAAGGACCTGGCGGTGCTTCATATCCCTCTAGAGGAGCCTGTTCTGTAA

>Bok011_clone8 (*Homo sapiens*)

ACAGTTAAATCAACAAAACTGCTCGCCAGAACACTACGAGCCACAGCTTAAAACTCAAAGGACCTGGCGGTGCTTCATATCCCTCTAGAGGAGCCTGTTCTGTAA

**BOK012**

>Bok012_clone1 (*Canis lupus*)

AAATAATTCGCCAGAGGACTACTAGCAATAGCTTAAAACTCAAAGGACTTGGCGGTGCTTTATATCCCTCTAGAGGAGCCTGTTCTATAATCGATAAACCCCG

>Bok012_clone2 (*Canis lupus*)

TAATTTTACAACAAAATAATTCGCCAGAGGACTACTAGCAATAGCTTAAAACTCAAAGGACTTGGCGGTGCTTTATATCCCTCTAGAGGAGCCTGTTCTATAATCGATAAACCCCG

>Bok012_clone3 (*Canis lupus*)

AATTTTACAACAAAATAATTCGCCAGAGGACTACTAGCAATAGCTTAAAACTCAAAGGACTTGGCGGTGCTTTATATCCCTCTAGAGGAGCCTGTTCTATAATCGATAAACCCCG

>Bok012_clone4 (*Canis lupus*)

ACAAAATAATTCGCCAGAGGACTACTAGCAATAGCTTAAAACTCAAAGGACTTGGCGGTGCTTTATATCCCTCTAGAGGAGCCTGTTCTATAATCGATAAACCCCG

>Bok012_clone5 (*Canis lupus*)

ATTTTACAACAAAATAATTCGCCAGAGGACTACTAGCAATAGCTTAAAACTCAAAGGACTTGGCGGTGCTTTATATCCCTCTAGAGGAGCCTGTTCTATAATCGATAAACCCCG

>Bok012_clone6 (*Canis lupus*)

ATTTTACAACAAAATAATTCGCCAGAGGACTACTAGCAATAGCTTAAAACTCAAAGGACTTGGCGGTGCTTTATATCCCTCTAGAGGAGCCTGTTCTATAATCGATAAACCCCG

**BOK013**

>Bok013_clone1 *(Bos* sp.)

ATTCGCCAGAGTACTACTAGCAACAGCTTAAAACTCAAAGGACTTGGCGGTGCTTTATATCCTTCTAGAGGAGCCTGTTCTATAAT

>Bok013_clone2 (*Bos* sp.)

ATTCGCCAGAGTACTACTAGCAACAGCTTAAAACTCAAAGGACTTGGCGGTGCTTTATATCCTTCTAGAGGAGCCTGTTCTATAAT

>Bok013_clone3 (*Homo sapiens*)

GCTCGCCAGAACACTACGAGCCACAGCTTAAAACTCAAAGGACCTGGCGGTGCTTCATATCCCTCTAGAGGAGCCTGTTCTGTA

>Bok013_clone4 (*Bos* sp.)

ATTCGCCAGAGTACTACTAGCAACAGCTTAAAACTCAAAGGACTTGGCGGTGCTTTATATCCTTCTAGAGGAGCCTGTTCTATAAT

>Bok013_clone5 (*Bos* sp*.*)

ATTCGCCAGAGTACTACTAGCAACAGCTTAAAACTCAAAGGACTTGGCGGTGCTTTATATCCTTCTAGAGGAGCCTGTTCTATAAT

>Bok013_clone6 (*Bos* sp.)

ATTCGCCAGAGTACTACTAGCAACAGCTTAAAACTCAAAGGACTTGGCGGTGCTTTATATCCTTCTAGAGGAGCCTGTTCTATAAT

>Bok013_clone7 (*Bos* sp.)

ATTCGCCAGAGTACTACTAGCAACAGCTTAAAACTCAAAGGACTTGGCGGTGCTTTATATCCTTCTAGAGGAGCCTGTTCTATAAT

>Bok013_clone8 (*Bos* sp.)

TTCGCCAGAGTACTACTAGCAACAGCTTAAAACTCAAAGGACTTGGCGGTGCTTTATATCCTTCTAGAGGAGCCTGTTCTATAATCG

**BOK014**

>Bok014_clone1 (*Homo sapiens*)

TCAACAAAACTGCTCGCCAGAGCACTACGAGCCACAGCTTAAAACTCAAAGGACCTGGCGGTGCTTCATACCCCTCTAGAGGAGCCTGTTCTGTAATCGATA

>Bok014_clone2 (*Homo sapiens*)

AATCAACAAAACTGCTCGCCAGAACACTACGAGCCACAGCTTAAAACTCAAAGGACCTGGCGGTGCTTCATATCCCTCTAGAGGAGCCTGTTCTGTAATCGATAAACCCCGATCAACCTCA

>Bok014_clone3 (*Homo sapiens*)

ACAAAACTGCTCGCCAGAACACTACGAGCCACAGCTTAAAACTCAAAGGACCTGGCGGTGCTTCATATCCCTCTAGAGGAGCCTGTTCTGTAATCGATAAACCCCGATCAACCTCACCACCTC

>Bok014_clone4 (*Canis lupus*)

ATTTTACAACAAAATAATTCGCCAGAGGACTACTAGCAATAGCTTAAAACTCAAAGGACTTGGCGGTGCTTTATATCCCTCTAGAGGAGCCTGTTCTATAATCGATAAACCCCGATAAACCTCACCACCT

>Bok014_clone5 (*Bos* sp.)

ACACAGATAATTACATAAACAAAATTATTCGCCAGAGTACTACTAGCAACAGCTTAAAACTCAAAGGACTTGGCGGTGCTTTATATCCTTCTAGAGGAGCCTGTTCTATAATCGATAAACCCCGATAAACCTCACCAAT

**BOK015**

> Bok015_clone1 (*Zenaida* sp*./ Columba livia*)

CTAGATGCTTATACTACTAAAGCATCCGCCTGAGGACTACGAGCACAAACGCTTAAAACTCTAAGGACTTGGCGGTGCCCTGAACCCACCTAGAGGAGCCTGTTCTATAATCGATGACCCACGATACACCCGACCACT

**BOK054**

>Bok054_clone1 (*Canis lupus*)

ACAAAATAATTCGCCAGAGGACTACTAGCAATAGCTTAAAACTCAAAGGACTTGGCGGTGCTTTATATCCCTCTAGAGGAGCCTGTTCTATAATCGATAAACCCCGAT

>Bok054_clone2 (*Canis lupus*)

AACAAAATAATTCGCCAGAGGACTACTAGCAATAGCTTAAAACTCAAAGGACTTGGCGGTGCTTTATATCCCTCTAGAGGAGCCTGTTCTATAATCG

>Bok054_clone3 (*Canis lupus*)

TTTTACAACAAAATAATTCGCCAGAGGACTACTAGCAATAGCTTAAAACTCAAAGGACTTGGCGGTGCTTTATATCCCTCTAGAGGAGCCTGTTCTATAATCGATAAACCCCGAT

>Bok054_clone4 (*Canis lupus*)

AAAATAATTCGCCAGAGGACTACTAGCAATAGCTTAAAACTCAAAGGACTTGGCGGTGCTTTATATCCCTCTAGAGGAGCCTGTTCTATAATCGATAAACCCCG

>Bok054_clone5 (*Canis lupus*)

AAATAATTCGCCAGAGGACTACTAGCAATAGCTTAAAACTCAAAGGACTTGGCGGTGCTTTATATCCCTCTAGAGGAGCCTGTTCTATAATCGATAAACCCCGATAAACCTC

**BOK056**

>Bok056_clone1 (*Canis lupus*)

AAATAATTCGCCAGAGGACTACTAGCAATAGCTTAAAACTCAAAGGACTTGGCGGTGCTTTATATCCCTCTAGAGG

>Bok056_clone2 (*Canis lupus*)

ACAACAAAATAATTCGCCAGAGGACTACTAGCAATAGCTTAAAACTCAAAGGACTTGGCGGTGCTTTATATCCCTCTAGAGGAGCCTGTTCTATAATCGATAAACCCCGATAAACCTCACCA

**BOK062**

>Bok062_clone1 (*Homo sapiens*)

AATCAACAAAACTGCTCGCCAGAACACTACGAGCCACAGCTTAAAACTCAAAGGACCTGGCGGTGCTTCATATCCCTCTAGAGGAGCCTGTTCTGTAATCGATAAACCCCGATCAACCTCA

>Bok062_clone2 (*Homo sapiens*)

AATCAACAAAACTGCTCGCCAGAACACTACGAGCCACAGCTTAAAACTCAAAGGACCTGGCGGTGCTTCATATCCCTCTAGAGGAGCCTGTTCTGTAATCGATAAACCCCGATCAACCTCA

>Bok062_clone3 (*Homo sapiens*)

ACAGTTAAATCAACAAAACTGCTCGCCAGAACACTACGAGCCACAGCTTAAAACTCAAAGGACCTGGCGGTGCTTCATATCCCTCTAGAGGAGCCTGTTCTGTAATCGATAAACCCCGATCAACC

>Bok062_clone4 (*Homo sapiens*)

AATCAACAAAACTGCTCGCCAGAACACTACGAGCCACAGCTTAAAACTCAAAGGACCTGGCGGTGCTTCATATCCCTCTAGAGGAGCCTGTTCTGTAATCGATAAACCCCGA

>Bok062_clone5 (*Homo sapiens*)

AATCAACAAAACTGCTCGCCAGAACACTACGAGCCACAGCTTAAAACTCAAAGGACCTGGCGGTGCTTCATATCCCTCTAGAGGAGCCTGTTCTGTAATCGATAAACCCCGATCAACCTCACCA

>Bok062_clone6 (*Homo sapiens*)

ACAAAACTGCTCGCCAGAACACTACGAGCCACAGCTTAAAACTCAAAGGACCTGGCGGTGCTTCATATCCCTCTAGAGGAGCCTGTTCTGTAATCGATAAACCCCGATCAACCTCA

>Bok062_clone7 (*Homo sapiens*)

ACAAAACTGCTCGCCAGAACACTACGAGCCACAGCTTAAAACTCAAAGGACCTGGCGGTGCTTCATATCCCTCTAGAGGAGCCTGTTCTGTAATCGATAAACCCCGA

>Bok062_clone8 (*Homo sapiens*)

TAAATCAACAAAACTGCTCGCCAGAACACTACGAGCCACAGCTTAAAACTCAAAGGACCTGGCGGTGCTTCATATCCCTCTAGAGGAGCCTGTTCTGTAATCGATAAACCCCGATCA

**BOK065**

>Bok065_clone1 (*Homo sapiens*)

ACAGTTAAATCAACAAAACTGCTCGCCAGAACACTACGAGCCACAGCTTAAAACTCAAAGGACCTGGCGGTGCTTCATATCCCTCTAGAGGAGCCTGTTCTGTAATCGATAAACCCCG

>Bok065_clone2 (*Homo sapiens*)

TAAACCTCAACAGTTAAATCAACAAAACTGCTCGCCAGAACACTACGAGCCACAGCTTAAAACTCAAAGGACCTGGCGGTGCTTCATATCCCTCTAGAGGAGCCTGTTCTGTAATCGATAAACCCCGATCAACCTCACCA

>Bok065_clone3 (*Homo sapiens*)

ACAGTTAAATCAACAAAACTGCTCGCCAGAACACTACGAGCCACAGCTTAAAACTCAAAGGACCTGGCGGTGCTTCATATCCCTCTAGAGGAGCCTGTTCTGTAATCGATAAACCCCGATCAACCTCACCACCTC

>Bok065_clone4 (*Homo sapiens*)

TAAATCAACAAAACTGCTCGCCAGAACACTACGAGCCACAGCTTAAAACTCAAAGGACCTGGCGGTGCTTCATATCCCTCTAGAGGAGCCTGTTCTGTAATCGATAAACCCCG

>Bok065_clone5 (*Homo sapiens*)

ACAGTTAAATCAACAAAACTGCTCGCCAGAACACTACGAGCCACAGCTTAAAACTCAAAGGACCTGGCGGTGCTTCATATCCCTCTAGAGGAGCCTGTTCTGTAATCGATAAACCCCG

>Bok065_clone6 (*Homo sapiens*)

ACAAAACTGCTCGCCAGAACACTACGAGCCACAGCTTAAAACTCAAAGGACCTGGCGGTGCTTCATATCCCTCTAGAGGAGCCTGTTCTGTAATCGATAAACCCCGATCAACCTCACC

>Bok065_clone7 (*Homo sapiens*)

AAACTGCTCGCCAGAACACTACGAGCCACAGCTTAAAACTCAAAGGACCTGGCGGTGCTTCATATCCCTCTAGAGGAGCCTGTTCTGTAATCGATAAACC

**BOK069**

>Bok069_clone1 (*Canis lupus*)

AAATAATTCGCCAGAGGACTACTAGCAATAGCTTAAAACTCAAAGGACTTGGCGGTGCTTTATATCCCTCTAGAGGAGCCTGTTCTATAATCGATAAACCCCGAT

>Bok069_clone2 (*Canis lupus*)

AAATAATTCGCCAGAGGACTACTAGCAATAGCTTAAAACTCAAAGGACTTGGCGGTGCTTTATATCCCTCTAGAGGAGCCTGTTCTATAATCGATAAACCCCGATAA

>Bok069_clone3 (*Canis lupus*)

TACAACAAAATAATTCGCCAGAGGACTACTAGCAATAGCTTAAAACTCAAAGGACTTGGCGGTGCTTTATATCCCTCTAGAGGAGCCTGTTCTATAATCGATAAACCCCG

**BOK072**

>Bok072_clone1 (*Bos* sp.)

CAGATAATTACATAAACAAAATTATTCGCCAGAGTACTACTAGCAACAGCTTAAAACTCAAAGGACTTGGCGGTGCTTTATATCCTTCTAGAGGAGCCTGTTCTATAATCGATAAACCCCGATAAACCTCACCA

**BOK077**

>Bok077_clone1 (*Bos* sp.)

AAAATTATTCGCCAGAGTACTACTAGCAACAGCTTAAAACTCAAAGGACTTGGCGGTGCTTTATATCCTTCTAGAGGAGCCTGTTCTATAATCGATAAACCCCGATAAAC

**BOK083**

>Bok083_clone1 (*Mus musculus*) *

TATTTGCCAGAGAACTACTAGCCATAGCTTAAAACTCAAAGGACTTGGCGGTACTTTATATCCATCTAGAGGAGCCTGTTCTATAATCGATAAACCCCGCTCTACCTCACCATCTCTTGCTAATTCAGCCTATATACCGCCATCTTCAGCAAACA

>Bok083_clone2 (*Mus musculus*) *

TATTTGCCAGAGAACTACTAGCCATAGCTTAAAACTCAAAGGACTTGGCGGTACTTTATATCCATCTAGAGGAGCCTGTTCTATAATCGATAAACCCCGCTCTACCTCACCATCTCTTGCTAATTCAGCCTATATACCGCCATCTTCAGCAAACA

>Bok083_clone3 (*Mus musculus*) *

TATTTGCCAGAGAACTACTAGCCATAGCTTAAAACTCAAAGGACTTGGCGGTACTTTATATCCATCTAGAGGAGCCTGTTCTATAATCGATAAACCCCGCTCTACCTCACCATCTCTTGCTAATTCAGCCTATATACCGCCATCTTCAGCAAACA

>Bok083_clone4 (*Mus musculus*) *

TATTTGCCAGAGAACTACTAGCCATAGCTTAAAACTCAAAGGACTTGGCGGTACTTTATATCCATCTAGAGGAGCCTGTTCTATAATCGATAAACCCCGCTCTACCTCACCATCTCTTGCTAATTCAGCCTATATACCGCCATCTTCAGCAAACA

>Bok083_clone5 (*Mus musculus*) *

TATTTGCCAGAGAACTACTAGCCATAGCTTAAAACTCAAAGGACTTGGCGGTACTTTATATCCATCTAGAGGAGCCTGTTCTATAATCGATAAACCCCGCTCTACCTCACCATCTCTTGCTAATTCAGCCTATATACCGCCATCTTCAGCAAACA

**BOK084**

>Bok084_clone1 (*Bos* sp.) *

TTATTCGCCAGAGTACTACTAGCAACAGCTTAAAACTCAAAGGACTTGGCGGTGCTTTATATCCTTCTAGAGGAGCCTGTTCTATAATCGATAAACCCCGATAAACCTCACCAATTCTTGCTAATACAGTCTATATACCGCCATCTTCAGCAAACA

**BOK103**

>Bok103_clone1 (*Homo sapiens*)

AAACTGCTCGCCAGAACACTACGAGCCACAGCTTAAAACTCAAAGGACCTGGCGGTGCTTCATATCCCTCTAGAGGAGCCTGTTCTGTAATCGATAAACCCCGATCAACCTCACC

>Bok103_clone2 (*Homo sapiens*)

AACCTCAACAGTTAAATCAACAAAACTGCTCGCCAGAACACTACGAGCCACAGCTTAAAACTCAAAGGACCTGGCGGTGCTTCATATCCCTCTAGAGGAGCCTGTTCTGTAATCGATAAACCCCG

>Bok103_clone3 (*Homo sapiens*)

ATCAACAAAACTGCTCGCCAGAACACTACGAGCCACAGCTTAAAACTCAAAGGACCTGGCGGTGCTTCATATCCCTCTAGAGGAGCCTGTTCTGTAATCGATAAACCCCG

>Bok103_clone4 (*Homo sapiens*)

ACAGTTAAATCAACAAAACTGCTCGCCAGAACACTACGAGCCACAGCTTAAAACTCAAAGGACCTGGCGGTGCTTCATATCCCTCTAGAGGAGCCTGTTCTGTAATCGATAAACCCCGATCAACCTCAC

>Bok103_clone5 (*Homo sapiens*)

ACAAAACTGCTCGCCAGAACACTACGAGCCACAGCTTAAAACTCAAAGGACCTGGCGGTGCTTCATATCCCTCTAGAGGAGCCTGTTCTGTAATCGATAA

>Bok103_clone6 (*Homo sapiens*)

ACAAAACTGCTCGCCAGAACACTACGAGCCACAGCTTAAAACTCAAAGGACCTGGCGGTGCTTCATATCCCTCTAGAGGAGCCTGTTCTGTAATCGATAAACCCCGATCAACCTC

**SUD006**

>Sud006_clone1 (*Sus scrofa*)

AATAGTTACATAACAAAACTATTCGCCAGAGTACTACTCGCAACTGCCTAAAACTCAAAGGACTTGGCGGTGCTTCACATCCACCTAGAGGAGCCTGTTCTATAATCGATAAACCCCGATAGACCTTACCA

>Sud006_clone2 (*Sus scrofa*)

ATAGTTACATAACAAAACTATTCGCCAGAGTACTACTCGCAACTGCCTAAAACTCAAAGGACTTGGCGGTGCTTCACATCCACCTAGAGGAGCCTGTTCTATAATCGATAAACCCCGATAGACCTTACCA

>Sud006_clone3 (*Sus scrofa*)

AATAGTTACATAACAAAACTATTCGCCAGAGTACTACTCGCAACTGCCTAAAACTCAAAGGACTTGGCGGTGCTTCACATCCACCTAGAGGAGCCTGTTCTATAATCGATAAACCCCGATAGACCTTACCAACC

>Sud006_clone4 (*Sus scrofa*)

AATAGTTACATAACAAAACTATTCGCCAGAGTACTACTCGCAACTGCCTAAAACTCAAAGGACTTGGCGGTGCTTCACATCCACCTAGAGGAGCCTGTTCTATAATCGATAAACCCCGATAGACCTTACCA

>Sud006_clone5 (*Sus scrofa*)

ATAGTTACATAACAAAACTATTCGCCAGAGTACTACTCGCAACTGCCTAAAACTCAAAGGACTTGGCGGTGCTTCACATCCACCTAGAGGAGCCTGTTCTATAATCGATAAACCCCGATAGACCTTACCA

>Sud006_clone6 (*Sus scrofa*)

AATAGTTACATAACAAAACTATTCGCCAGAGTACTACTCGCAACTGCCTAAAACTCAAAGGACTTGGCGGTGCTTCACATCCACCTAGAGGAGCCTGTTCTATAATCGATAAACCCCGATAGACCTTACCAACC

>Sud006_clone7 (*Sus scrofa*)

ATAGTTACATAACAAAACTATTCGCCAGAGTACTACTCGCAACTGCCTAAAACTCAAAGGACTTGGCGGTGCTTCACATCCACCTAGAGGAGCCTGTTCTATAATCGATAAACCCCGATAGACCTTACCAACC

**SUD007**

>Sud007_clone1 (*Homo sapiens*)

ACTCGAATAGTTAGATCAACAAAACTGTTCGCCAGAACACTACGAGCCACAGCTTAAAACTCAAAGGACCTGGCGGTGCTTCATATCCCTCTAGAGGAGCCTGTTCTGTAATCGATAAACCCCGATCAACCTCACCAC

>Sud007_clone2 (*Homo sapiens*)

CAGTTAAATCAACAAAACTGCTCGCCAGAACACTACGAGCCACAGCTTAAAACTCAAAGGACCTGGCGGTGCTTCATATCCCTCTAGAGGAGCCTGTTCTGTTATCGATAAACCCCGATCAACCTCACCAC

>Sud007_clone3 (*Homo sapiens*)

CAGTTAAATCAACAAAACTGCTCGCCAGAACACTACGAGCCACAGCTTAAAACTCAAAGGACCTGGCGGTGCTTCATATCCCTCTAGAGGAGCCTGTTCTGTAATCGATAAACCCCGATCAACCTCAC

**SUD010**

>Sud010_clone1 (*Canis lupus*)

AACATAGATAATTTTACAACAAAATAATTCGCCAGAGGACTACTAGCAATAGCTTAAAACTCAAAGGACTTGGCGGTGCTTTATATCCCTCTAGAGGAGCCTGTTCTATAATCGATAAACCCCGATAAACCTCACCAC

>Sud010_clone2 (*Mus musculus*) *

GCCAGAGAACTACTAGCCATAGCTTAAAACTCAAAGGACTTGGCGGTACTTTATATCCATCTAGAGGAGCCTGTTCTATAATCGATAAACCCCGCTCTACCTCACCATCTCTTGCTAAT

**SUD011**

>Sud011_clone1 (*Pecari tajacu*)

CTAAATAATCGACCAACAAGATTATTCGCCAGAGTACTACTAGCAACAGCCTAAAACTCAAAGGACTTGACGGTGCTTCATATCCATCTAGAGGAGCCTGTTCTATAATCGATAAACCCCGATAAACCTCACCA

**SUD013**

>Sud013_clone1 (*Sus scrofa*)

CAAATAGTTACATAACAAAACTATTCGCCAGAGTACTACTCGCAACTGCCTAAAACTCAAAGGACTTGGCGGTGCTTCACATCCACCTAGAGGAGCCTGTTCTATAATCGATAAACCCCGATAGACCTTACCAACC

>Sud013_clone2 (*Sus scrofa*)

CCAAATAGTTACATAACAAAACTATTCGCCAGAGTACTACTCGCAACTGCCTAAAACTCAAAGGACTTGGCGGTGCTTCACATCCACCTAGAGGAGCCTGTTCTATAATCGATAAACCCCGATAGACCTTACCA

>Sud013_clone3 (*Sus scrofa*)

CCAAATAGTTACATAACAAAACTATTCGCCAGAGTACTACTCGCAACTGCCTAAAACTCAAAGGACTTGGCGGTGCTTCACATCCACCTAGAGGAGCCTGTTCTATAATCGATAAACCCCGATAGACCTTACCA

>Sud013_clone4 (*Sus scrofa*)

AATAGTTACATAACAAAACTATTCGCCAGAGTACTACTCGCAACTGCCTAAAACTCAAAGGACTTGGCGGTGCTTCACATCCACCTAGAGGAGCCTGTTCTATAATCGATAAACCCCGATAGACCTTACCAACC

>Sud013_clone5 (*Sus scrofa*)

CAAATAGTTACATAACAAAACTATTCGCCAGAGTACTACTCGCAACTGCCTAAAACTCAAAGGACTTGGCGGTGCTTCACATCCACCTAGAGGAGCCTGTTCTATAATCGATAAACCCCGATAGACCTTACCA

>Sud013_clone6 (*Sus scrofa*)

AATAGTTACATAACAAAACTATTCGCCAGAGTACTACTCGCAACTGCCTAAAACTCAAAGGACTTGGCGGTGCTTCACATCCACCTAGAGGAGCCTGTTCTATAATCGATAAACCCCGATAGACCTTACCAACC

**SUD015**

>Sud015_clone1 (*Homo sapiens*)

AAACTGCTCGCCAGAACACTACGAGCCACAGCTTAAAACTCAAAGGACCTGGCGGTGCTTCATATCCCTCTAGAGGAGCCTGTTCTGTA

>Sud015_clone2 (*Homo sapiens*)

AACTGCTCGCCAGAACACTACGAGCCACAGCTTAAAACTCAAAGGACCTGGCGGTGCTTCATATCCCTCTAGAGGAGCCTGTTCTGTAATCGATA

**SUD017**

>Sud017_clone1 (*Sus scrofa*)

CCCAAATAGTTACATAACAAAACTATTCGCCAGAGTACTACTCGCAACTGCTTAAAACTCAAAGGACTTGGCGGTGCTTCACATCCACCTAGAGGAGCCTGTTCTATAATCGATAAACCCTGATAGACCTTACCAACC

>Sud017_clone2 (*Homo sapiens*) **

ACTCTAAACTCAAATAGTTAGATCAAAAAAACTGTTAGCCAGAACACTACAAGCAACAGCTTAAAACTCAAAGGACTTGGCGGTGCTTTATATCCCTCTAAAGGAGCCTGTTCTGTAATCGATAAACCCCGATCAACCTCACCACC

>Sud017_clone3 (*Sus scrofa*)

ACCCAAATAGTTACATAACAAAACTATTCGCCAGAGTACTACTCGCAACTGCCTAAAACTCAAAGGACTTGGCGGTGCTTCACATCCACCTAGAGGAGCCTGTTCTATAATCGATAAACCCCGATAGACCTTACCA

>Sud017_clone4 (*Sus scrofa*)

ACCCAAATAGTTACATAACAAAACTATTCGCCAGAGTACTACTCGCAACTGCCTAAAACTCAAAGGACTTGGCGGTGCTTCACATCCACCTAGAGGAGCCTGTTCTATAATCGATAAACCCCGATAGACCTTACCA

>Sud017_clone5 (*Sus scrofa*)

CAAATAGTTACATAACAAAACTATTCGCCAGAGTACTACTCGCAACTGCCTAAAACTCAAAGGACTTGGCGATGCTTCACATCCACCTAGAGGAGCCTGTTCTATAATCGATAAACCCCGATAGACCTTACCA

>Sud017_clone6 (*Sus scrofa*) ACCCAAATAGTTACATAACAAAACTATTCGCCAGAGTACTACTCGCAACTGCCTAAAACTCAAAGGACTTGGCGGTGCTTCACATCCACCTAGAGGAGCCTGTTCTATAATCGATAAACCCCGATAGACCTTACCA

>Sud017_clone7 (*Homo sapiens*) ** ACTCTAAACTCAAATAGTTAGATCAAAAAAACTGTTCGCCAGAACACTACAAGCAACAGCTTAAAACTCAAAGGACTTGGCGGTGCTTTATATCCCTCTAAAGGAGCCTGTTCTGTAATCGATAAACCCCGATCAACCTCACCACC

**SUD020**

>Sud020_clone1 (*Canis lupus*)

TAGATAATTTTACAACAAAATAATTCGCCAGAGGACTACTAGCAATAGCTTAAAACTCAAAGGACTTGGCGGTGCTTTATATCCCTCTAGAGGAGCCTGTTCTATAATCGATAAACCCCGATAAACCTCACCAC

>Sud020_clone2 (*Canis lupus*)

TAGATAATTTTACAACAAAATAATTCGCCAGAGGACTACTAGCAATAGCTTAAAACTCAAAGGACCTGGCGGTGCTTTATATCCCTCTAGAGGAGCCTGTTCTATAATCGATAAACCCCGATAAACCTCACCACCT

>Sud020_clone3 (*Canis lupus*)

TAGATAATTTTACAACAAAATAATTCGCTAGAGGACTACTAGCAATAGTTTAAAACTCAAAGGACTTGGCGGCGCCTTATATCCCTCTAGAGGAGCCTGTTCTATAATCGATAAACCCCGATAAACCTCACCAC

>Sud020_clone4 (*Canis lupus*)

ATTTTACAACAAAATAATTCGCCAGAGGGCTACTAGCAATAGCTTAAAACTCAAAGGACCTGGCGGTGCTTTATATCCCTCTAGAGGAGCCTGTTCTATAATCGATAAACCCCGATAAACCTCACCACCT

>Sud020_clone5 (*Canis lupus*)

TAGATAATTTTACAACAAAATAATTCGCCAGAGGGCTACTAGCAATAGCTTAAAACTCAAAGGACCTGGCGGTGCTTTATATCCCTCTAGAGGAGCCTGTTCTATAATCGATAAACCCCGATAAACCTCACCACCT

**SUD023**

>Sud023_clone1 (*Homo sapiens*) **

AACTCTAGTAGTTACATTAACAAAACCATTCGTCAGAATACTACGAGCAACAGCTTAAAACTCAAAGGACCTGGCAGTTCTTTATATCCCTCTAGAGAAGCCTGTTCTATAAGCGATAAACCCGATACACCTCACCAC

>Sud023_clone2 (*Mus musculus*)

ATTTAACAAAACTATTTGCCAGAGAACTACTAGCCATAGCTTAAAACTCAAAGGACTTGGCGGTACTTTATATCCATCTAGAGGAGCCTGTTCTATAATCGATAAACCCCGCTCTACCTC

>Sud023_clone3 (*Mus musculus*)

AATAATTAAATTTAACAAAACTATTTGCCAGAGAACTACTAGCCATAGCTTAAGACTCAAAGGACTTGGCGGTACTTTATATCCATCTAGAGGAGCCTGTTCTATAATCGATAAACCCCGCTCTACCTCACCATCTCT

**SUD025**

>Sud025_clone1 (*Mus musculus*)

ATTAAATTTAACAAAACTATTTGCCAGAGAACTACTAGCCATAGCTTAAAACTCAAAGGACTTGGCGGTACTTTATATCCATCTAGAGGAGCCTGTTCTATAATCGATAAACCCCGCTCTACCTCACCA

>Sud025_clone2 (*Mus musculus*) *

AAACTATTTGCCAGAGAACTACTAGCCATAGCTTAAAACTCAAAGGACTTGGCGGTACTTTATATCCATCTAGAGGAGCCTGTTCTATAATCGATAAACCCCGCTCTACCTCACCATCTCTTGCTAATTCAGCCTATATACCGCCATCTTCAGCAAAC

**SUD026**

>SUD026_clone1 (*Zenaida/Columba* sp*.*)

ATCTAGATGCTTATACTACTAAAGCATCCGCCTGAGGACTACGAGCACAAACGCTTAAAACTCTAAGGACTTGGCGGTGCCCTAAACCCACCTAGAGGAGCCTGTTCTATAA

>SUD026_clone2 (*Columba* sp*.*)

CGCCTGAGGACTACGAGCACAAACGCTTAAAACTCTAAGGACTTGGCGGTGCCCTAAACCCACCTAGAGGAGCCTGTTCTATAA

>SUD026_clone3 (*Zenaida/Columba* sp*.*)

ATCTAGATGCTTATACTACTAAAGCATCCGCCTGAGGACTACGAGCACAAACGCTTAAAACTCTAAGGACTTGGCGGTGCCCTAAACCCACCTAGAGGAGCCTGTTCTATAA

>SUD026_clone4 (*Zenaida/Columba* sp*.*)

ATCTAGATGCTTATACTACTAAAGCATCCGCCTGAGGACTACGAGCACAAACGCTTAAAACTCTAAGGACTTGGCGGTGCCCTAAACCCACCTAGAGGAGCCTGTTCTATAATCGATAACCCACGATACACCCGAC

**SUD032**

>Sud032_clone1 (*Homo sapiens*)

TCAACAAAACTGCTCGCCAGAACACTACGAGCCACAGCTTAAAACTCAAAGGACCTGGCGGTGCTTCATATCCCTCTAGAGGAGCCTGTTCTGTAATCGATAAACCCCGATCA

**SUD033**

>Sud033_clone1 (*Homo sapiens*)

GCTCGCCAGAACACTACGAGCCACAGCTTAAAACTCAAAGGACCTGGCGGTGCTTCATATCCCTCTAGAGGAGCCTGTTCTGTAATCGATAAA

>Sud033_clone2 (*Homo sapiens*)

GCTCGCCAGAACACTACGAGCCACAGCTTAAAACTCAAAGGACCTGGCGGTGCTTCATATCCCTCTAGAGGAGCCTGTTCTGTAATCG

>Sud033_clone3 (*Homo sapiens*)

GCTCGCCAGAACACTACGAGCCACAGCTTAAAACTCAAAGGACCTGGCGGTGCTTCATATCCCTCTAG

>Sud033_clone4 (*Homo sapiens*)

CGCCAGAACACTACGAGCCACAGCTTAAAACTCAAAGGACCTGGCGGTGCTTCATATCCCTCTAG

>Sud033_clone5 (*Homo sapiens*)

AATCAACAAAACTGCTCGCCAGAACACTACGAGCCACAGCTTAAAACTCAAAGGACCTGGCGGTGCTTCATATCCCTCTAGAGGAGCCTGTTCTGTAATCG

>Sud033_clone6 (*Homo sapiens*)

GTTAAATCAACAAAACTGCTCGCCAGAACACTACGAGCCACAGCTTAAAACTCAAAGGACCTGGCGGTGCTTCATATCCCTCTAGAGGAGCCTGTTCTGTAATC

**SUD036**

>Sud036_clone1 (*Artibeus lituratus*)

AAGAGTCCTCCCAACAAGACTCTTCGCCAGAGTACTACTAGCCAAAGCTTAAAACTCAAGGGACTTGGCGGTGCTTCATATCCCTCTAGAGGAGCCTGTTCTATAATCGATAAACCCCGATCAACCTCACCAA

**SUD042**

>Sud042_clone1 (*Homo sapiens*)

ACGGTTAAATCAACAAAACTGCTCGCCAGAACACTACGAGCCACAGCTTAAAACTCAAAGGACCTGGCGGTGCTTCATATCCCTCTAGAGGAGCCTGTTCTGTAATCGATAA

>Sud042_clone2 (*Homo sapiens*) **

ATAGTTAGATCAACAAAACTGTTCACCAGAACACTACAAGCAACAGCTTAAAACTCAAAGGACTTGGCGGTACTTTATATCCCTCTAAAGGAGGCTGTTCTATAATCGATAAACCCCAA

>Sud042_clone3 (*Homo sapiens*)

ACGGTTAAATCAACAAAACTGCTCGCCAGAACACTACGAGCCACAGCTTAAAACTCAAAGGACCTGGCGGTGCTTCATATCCCTCTAGAGGAGCCTGTTCTGTAATCGATAAACCCCGATCAACCTCACCA

>Sud042_clone4 (*Homo sapiens*)

ACAGTTAAATCAACAAAACTGCTCGCCAGAACACTACGAGCCACAGCTTAAAACTCAAAGGACCTGGCGGTGCTTCATATCCCCCTAGAGGAGCCTGTTCTGTAATCGATAAACCCCGATCAACCTCACCAC

**SUD043**

>Sud043_clone1 (*Homo sapiens*) **

AAAGTCGAATAGTTACATTAACAAAACCATTCGCCAGACTACTACAAGCAACAGCTTAAAACTCAAAGGACTTGGCGGTGCCTTACATCCCTCTAGAGGAGCCTGTTCTATAGTAGACAAACCCCGATACAACTCACCATC

>Sud043_clone2 (*Homo sapiens*)

ACAGTTAAATCAACAAAACTGCTCGCCAGAACACTACGAGCCACAGCTTAAAACTCAAAGGACCTGGCGGTGCTTCATATCCCTCTAGAGGAGCCTGTTCTGTAATCGATAAACCCCGATCAACCTCA

>Sud043_clone3 (*Homo sapiens*) **

ACTCTAAACTCAAATAGTTAGATCAAAAAAACTGTTCGCCAGAACACTACAAGCAACAGCTTAAAACTCAAAGGACTTGGCGGTGCTTTATATCCCTCTAAAGGAGCCTGTTCTATAATCGATAAACCCCAATTTACCTCAC

>Sud043_clone4 (*Homo sapiens*) **

AGTCGAATAGTTACATTAACAAAACCATTCGCCAGACTACTACAAGCAACAGCTTAAAACTCAAAGGACTTGGCGGTGCCTTACATCCCTCTAGAGGAGCCTGTTCTATAGTAGACAAACCCCGATACAACTCAC

>Sud043_clone5 (*Canis lupus*)

TAGATAATTTTACAACAAAATAATTCGCCAGGGGACTACTAGCAATAGCTTAAAACTCAAAGGACTTGGCGGTGCTTTATATCCCTCTAGAGGAGCCTGTTCTATAATCGATAAACCCCGATAAACCTCACCAC

>Sud043_clone6 (*Homo sapiens*)

CAACAGTTAAATCAACAAAACTGCTCGCCAGAACACTACGAGCCACAGCTTAAAACTCAAAGGACCTGGCGGTGCTTCATATCCCTCTAGAGGAGCCTGTTCTGTAATCGATAAACCCCGATCAACCTCAC

**SUD045**

>Sud045_clone_1 (*Canis lupus*)

TTCGCCAGAGGACTACTAGCAATAGCTTAAAACTCAAAGGACTTGGCGGTGCTTTATATCCCTCTAGAGGAGCCTGTTCTATAATCGATAA

**SUD047**

>Sud047_clone1 (*Felis catus*)

TAGATAGTTATCCTAAACAAAACTATCCGCCAGAGAACTACTAGCAATAGCTTAAAACTCAAAGGACTTGGCGGTGCTTTACATCCCTCTAGAGGAGCCTGTTCTATAATCGATAAACCCCGATATACCTCA

>Sud047_clone2 (*Felis catus*)

AGATAGTTATCCTAAACAAAACTATCCGCCAGAGAACTACTAGCAATAGCTTAAAACTCAAAGGACTTGGCGGTGCTTTACATCCCTCTAGAGGAGCCTGTTCTATAATCGATAAACCCCGATATACCTCA

>Sud047_clone3 (*Felis catus*)

AGTTATCCTAAACAAAACTATCCGCCAGAGAACTACTAGCAATAGCTTAAAACTCAAAGGACTTGGCGGTGCTTTACATCCCTCTAGAGGAGCCTGTTCTATAATCGATAAACCCCGATATACCTCACCA

>Sud047_clone4 (*Felis catus*)

CCTAAACTTAGATAGTTATCCTAAACAAAACTATCCGCCAGAGAACTACTAGCAATAGCTTAAAACTCAAAGGACTTGGCGGTGCTTTACATCCCTCTAGAGGAGCCTGTTCTATAATCGATAAACCCCGATATACCTCACCA

>Sud047_clone5 (*Felis catus*)

TATCCTAAACAAAACTATCCGCCAGAGAACTACTAGCAATAGCTTAAAACTCAAAGGACTTGGCG

>Sud047_clone6 (*Felis catus*)

TAGATAGTTATCCTAAACAAAACTATCCGCCAGAGAACTACTAGCAATAGCTTAAAACTCAAAGGACTTGGCGGTGCTTTACATCCCTCTAGAGGAGCCTGTTCTATAATCGATAAACCCCGATATACCTCA

>Sud047_clone7 (*Felis catus*)

ACAAAACTATCCGCCAGAGAACTACTAGCAATAGCTTAAAACTCAAAGGACTTGGCGGTGCTTTACATCCCTCTAGAGGAGCCTGTTCTATAATCGATAAACCCCGATATACCTCA

>Sud047_clone8 (*Felis catus*)

GATAGTTATCCTAAACAAAACTATCCGCCAGAGAACTACTAGCAATAGCTTAAAACTCAAAGGACTTGGCGGTGCTTTACATCCCTCTAGAGGAGCCTGTTCTATAATCGATAAACCCCGATATACCTCA

**SUD058**

>Sud058_clone1 (*Homo sapiens*)

ACAGTTAAATCAACAAAACTGCTCGCCAGAACACTACGAGCCACAGCTTAAAACTCAAAGGACCTGGCGGTGCTTCATATCCCTCTAGAGGTGCCTGTTCTGTAATCTATAAACCCCGATCTACCTCACCAC

>Sud058_clone2 (*Homo sapiens*)

CAAAACTGCTCGCCAGAACACTACGAGCCACAGCTTAAAACTCAAAGGACCTGGCGGTGCTTCATATCCCTCTAGAGGTGCCTGTTCTGTAATCTATAAACCCCGATCTACCTCACCAC

**SUD073**

>Sud073_clone1 (*Homo sapiens*)

ACAGTTAAATCAACAAAACTGCTCGCCAGAACACTACGAGCCACAGCTTAAAACTCAAAGGACCTGGCGGTGCTTCATATCCCTCTAGAGGAGCCTGTTCTGTAATCGATAAACCCCGATCAACCTCA

>Sud073_clone2 (*Homo sapiens*)

ACAGTTAAATCAACAAAACTGCTCGCCAGAACACTACGAGCCACAGCTTAAAACTCAAAGGACCTGGCGGTGCTTCATATCCCTCTAGAGGAGCCTGTTCTGTAATCGATAAACCCCGATCAACCTCACCACCTC

>Sud073_clone3 (*Homo sapiens*)

ACAGTTAAATCAACAAAACTGCTCGCCAGAACACTACGAGCCACAGCTTAAAACTCAAAGGACCTGGCGGTGCTTCATATCCCTCTAGAGGAGCCTGTTCTGTAATCGATAAACCCCGATCAACCTCACCA

>Sud073_clone4 (*Homo sapiens*)

AATCAACAAAACTGCTCGCCAGAACACTACGAGCCACAGCTTAAAACTCAAAGGACCTGGCGGTGCTTCATATCCCTCTAGAGGAGCCTGTTCTGTAATCGATAAACCCCGATCAACCTCA

>Sud073_clone5 (*Homo sapiens*)

ACAGTTAAATCAACAAAACTGCTCACCAGAACACTACGAGCCACAGCTTAAAACTCAAAGGACCTGGCGGTGCTTCATATCCCTCTAGAGGAGCCTGTTCTGTAATCGATAAACCCCGATCAACCTCACCA

>Sud073_clone6 (*Homo sapiens*)

ACAGTTAAATCAACAAAACTGCTCGCCAGAACACTACGAGCCACAGCTTAAAACTCAAAGGACCTGGCGGTGCTTCATATCCCTCTAGAGGAGCCTGTTCTGTAATCGATAAACCCCGATCAACCTCACCAC

**SUD079**

>Sud079_clone_1 (*Homo sapiens*)

ACAGTTAAATCAACTAAACTGCTCGCCAGAACACTACGAGCCACAGCTTAAAACTCAAAGGACCTGGCGGTGCTTCATATCCCTCTAGAGGAGCCTGTTCTGTAATCGATAAACCCCGATCAACCTCACCAC

**SUD080**

>Sud080_clone1 (*Homo sapiens*)

ACAAAACTGCTCGCCAGAACACTACGAGCCACAGCTTAAAACTCAAAGGACCTGGCGGTGCTTCATATCCCTCTAGAGGAGCCTGTTCTGTAATCGATAAACCC

>Sud080_clone2 (*Homo sapiens*)

ACAAAACTGCTCGCCAGAACACTACGAGCCACAGCTTAAAACTCAAAGGACCTGGCGGTGCTTCATATCCCTCTAGAGGAGCCTGTTCTGTAATCGATAAACCC

>Sud080_clone3 (*Homo sapiens*)

ACAAAACTGCTCGCCAGAACACTACGAGCCACAGCTTAAAACTCAAAGGACCTGGCGGTGCTTCATATCCCTCTAGAGGAGCCTGTTCTGTAATCGATAAACCC

>Sud080_clone4 (*Homo sapiens*)

ACAAAACTGCTCGCCAGAACACTACGAGCCACAGCTTAAAACTCAAAGGACCTGGCGGTGCTTCATATCCCTCTAGAGGAGCCTGTTCTGTAATCGATAAACCC

>Sud080_clone5 (*Homo sapiens*)

ACAAAACTGCTCGCCAGAACACTACGAGCCACAGCTTAAAACTCAAAGGACCTGGCGGTGCTTCATATCCCTCTAGAGGAGCCTGTTCTGTAATCGATAAACCC

>Sud080_clone6 (*Homo sapiens*)

ACAAAACTGCTCGCCAGAACACTACGAGCCACAGCTTAAAACTCAAAGGACCTGGCGGTGCTTCATATCCCTCTAGAGGAGCCTGTTCTGTAATCGATAAACCC

>Sud080_clone7 (*Homo sapiens*)

ACAAAACTGCTCGCCAGAACACTACGAGCCACAGCTTAAAACTCAAAGGACCTGGCGGTGCTTCATATCCCTCTAGAGGAGCCTGTTCTGTAATCGATAAACCC

**SUD082**

>Sud082_clone1 (*Homo sapiens*)

GTTAAATCAACAAAACTGCTCGCCAGAACACTACGAGCCACAGCTTAAAACTCAAAGGACCTGGCGGTGCTTCATATCCCTCTAGAGGAGCCTGTTCTGTAATCGA

>Sud082_clone2 (*Homo sapiens*)

GTTAAATCAACAAAACTGCTCGCCAGAACACTACGAGCCACAGCTTAAAACTCAAAGGACCTGGCGGTGCTTCATATCCCTCTAGAGGAGCCTGTTCTGTAATCGA

>Sud082_clone3 (*Homo sapiens*)

GTTAAATCAACAAAACTGCTCGCCAGAACACTACGAGCCACAGCTTAAAACTCAAAGGACCTGGCGGTGCTTCATATCCCTCTAGAGGAGCCTGTTCTGTAATCGA

>Sud082_clone4 (*Homo sapiens*)

GTTAAATCAACAAAACTGCTCGCCAGAACACTACGAGCCACAGCTTAAAACTCAAAGGACCTGGCGGTGCTTCATATCCCTCTAGAGGAGCCTGTTCTGTAATCGA

>Sud082_clone5 (*Homo sapiens*)

GTTAAATCAACAAAACTGCTCGCCAGAACACTACGAGCCACAGCTTAAAACTCAAAGGACCTGGCGGTGCTTCATATCCCTCTAGAGGAGCCTGTTCTGTAATCGA

**SUD085**

>Sud085_clone1 (*Homo sapiens*)

AAAACTGCTCGCCAGAACACTACGAGCCACAGCTTAAAACTCAAAGGACCTGGCGGTGCTTCATATCCCTCTAGAGGAGCCTGT

**SUD086**

>Sud086_clone1 (*Felis catus*)

CCTAAACTTAGATAGTTATCTTAAACAAAACTATCCGCCAGAGAACTACTAGCAATAGCTTAAAACTCAAAGGACTTGGCGGTGCTTTACATCCCTCTAGAGGAGCCTGTTCTATAATCGATAAACCCCGATATACCTCACCA

**SUD087**

>Sud087_clone1 (*Homo sapiens*)

CTCAACAGTTAAATCAACAAAACTGCTCGCCAGAACACTACGAGCCACAGCTTAAAACTCAAAGGACCTGGCGGTGCTTCATATCCCTCTAGAGGAGCCTGTTCTGTAATCGATAAACCCCGATCAACCTCAC

>Sud087_clone2 (*Homo sapiens*)

CTCAACAGTTAAATCAACAAAACTGCTCGCCAGAACACTACGAGCCACAGCTTAAAACTCAAAGGACCTGGCGGTGCTTCATATCCCTCTAGAGGAGCCTGTTCTGTAATCGATAAACCCCGATCAACCTCAC

**SUD091**

>Sud091_clone1 (*Rana* sp*.*)

ACAATTTATTTACACCCACCAGCGCCAGGGAACTACGAGCAATGCTTAAAACCCAAAGGATTTGACGGTGTCCCACCCAGCTAGAGGAGCCTGTTCTATAATCGATGATCCCCGCTACACCCCAC

>Sud091_clone2 (*Rana* sp.)

ACAATTTATTTACACCCACCAGCGCCAGGGAACTACGAGCAATGCTTAAAACCCAAAGGATTTGACGGTGTCCCACCCAGCTAGAGGAGCCTGTTCTATAATCGATGATCCCCGCTACACCCCAC

>Sud091_clone3 (*Homo sapiens*) **

AGCTCTAGTAGTTACATTAACAAAACCATTCGTCAGAATACTACGAGCAACAGCTTAAAACTCAAAGGACCTGGCAGTTCTTTATATCCCTCTAGAGAAGCCTGTTCTATAAGCGATAAACCCGATACACCTCACCACCTC

>Sud091_clone4 (*Homo sapiens*)

CTCAACAGTTAAATCAACAAAACTGCTCGCCAGAACACTACGAGCCACAGCTTAAAACTCAAAGGACCTGGCGGTGCTTCATATCCCTCTAGAGGAGCCTGTTCTGTAATCGATAAACCCCGATCAACCTCA

**SUD094**

>Sud094_clone1 (*Coendou* sp.)

AAAAATTTTTGCCAGAGAACTACTAGCAACAGCTTAAAACTCAAAGGACTTGACGGTGCTTACACCCACCTAGAGGAGCCTGTTCTGTAATCGATAAACCCCGATCTACCTCACCGT

**SUD095**

>Sud095_clone1 (*Homo sapiens*)

GCTCGCCAGAACACTACGAGCCACAGCTTAAAACTCAAAGGACCTGGCGGTGCTTCATATCCCTCTAGAGGAGCCT

**SUD099**

>Sud099_clone1 (*Sciurus* sp*.*)

CCTAAACATAAATGTTCAACTAACAAGAACATTCGCCAGAGAACTACTAGCCACTGCTTAAAACTCAAAGGACTTGGCGGTGCTTTATACCCCTCTAGAGGAGCCTGTTCTATAATCGATAAACCCCGTTAAACCTGACCACTC

>Sud099_clone2 (*Sciurus* sp.)

CCTAAACATAAATGTTCAACTAACAAGAACATTCGCCAGAGAACTACTAGCCACTGCTTAAAACTCAAAGGACTTGGCGGTGCTTTATACCCCTCTAGAGGAGCCTGTTCTATAATCGACAAACCCCGTTAAACCTGACCACTCTT

>Sud099_clone3 (*Sciurus* sp.)

TAAATGTTCAACTAACAAGAACATTCGCCAGAGAACTACTAGCCACTGCTTAAGACTCAAAGGACTTGGCGGTGCTTTATACCCCTCTAGAGGAGCCTGTTCTATAATCGATAAACCCCGTTAA

**SUD103**

>Sud103_clone1 (*Felis catus*)

GATAGTTATCCTAAACAAAACTATCCGCCAGAGAACTACCAGCAACAGCTTAGAACTCAAAGGACTTGGCGGTGCTTTACATCCCTCTAGAGGAGCCTGTTCTATAATCGATAAACCCCGA

>Sud103_clone2 (*Felis catus*)

GATAGTTATCCTAAACAAAACTATCCGCCAGAGAACTACCAGCAACAGCTTAGAACTCAAAGGACTTGGCGGTGCTTTACATCCCTCTAGAGGAGCCTGTTCTATAATCGATAAACCCCGA

>Sud103_clone3 (*Homo sapiens*)

AACAAAACTGCTCGCCAGAACACTACGAGCCACAGCTTAAAACTCAAAGGACCTGGCGGTGCTTCATATCCCTCTAGAGGAGCCTGTTCTGTAATCGATAAACCCCGA

**SUD106**

>Sud106_clone1 (*Canis lupus*)

GATAATTTTACAACAAAATAATTCGCCAGAGGACTACTAGCAATAGCTTAAAACTCAAAGGACTTGGCGGTGCTTTATATCCCTCTAGAGGAGTCTGTTCTATAATCGATAAACCCCGATAAACCTCACCACC

>Sud106_clone2 (*Canis lupus*)

GATAATTTTACAACAAAATAATTCGCCAGAGGACTACTAGCAATAGCTTAAAACTCAAAGGACTTGGCGGTGCTTTATATCCCTCTAGAGGAGCCTGTTCTATAATCGATAAACCCCGATAAACCTCACCACC

>Sud106_clone3 (*Canis lupus*)

GATAATTTTACAACAAAATAATTCGCCAGAGGACTACTAGCAATAGCTTAAAACTCAAAGGACTTGGCGGTGCTTTATATCCCTCTAGAGGAGCCTGTTCTATAATCGATAAACCCCGATAAACCTCACCACC

**SUD109**

>Sud109_clone1 (*Meleagris gallopavo*)

TGATACTAATATACTCACGTATCCGCCTGAGAACTACGAGCACAAACGCTTAAAACTCTAAGGACTTGGCGGTGCCCTAAACCCACCTAGAGGAGCCTGTTCTGTAATCGATAATCCACGA

>Sud109_clone2 (*Meleagris gallopavo*)

TGATACTAATATACTCACGTATCCGCCTGAGAACTACGAGCACAAACGCTTAAAACTCTAAGGACTTGGCGGTGCCCCAAACCCACCTAGAGGAGCCTGTTCTATAATCGATAATCCACGA

>Sud109_clone3 (*Meleagris gallopavo*)

TGATACTAATATACTCACGTATCCGCCTGAGAACTACGAGCACAAACGCTTAAAACTCTAAGGACTTGGCGGTGCCCTAAACCTACCTAGAGGAGCCTGTTCTGTAATCGATAATCCACGA

>Sud109_clone4 (*Meleagris gallopavo*)

TGATACTAATATACTCACGTATCCGCCTGAGAACTACGAGCACAAACGCTTAAAACTCTAAGGACTTGGCGGTGCCCTAAACCCACCTAGAGGAGCCTGTTCTGTAATCGATAATCCACGA

**SUD110**

>Sud110_clone1 (*Meleagris gallopavo*)

AATATACTCACGTATCCGCCTGAGAACTACGAGCACAAACGCTTAAAACTCTAAGGACTTGGCGGTGCCCTAAACCCACCTAGAGGAGCCTGTTCTGTAATCGATAATCCACGATCCACCCAACCACCTCT

>Sud110_clone2 (*Homo sapiens*)

ACAGTTAAATCAACAAAACTGCTCGCCAGAACACTACGAGCCACAGCTTAAAACTCAAAGGACCTGGCGGTGCTTCATATCCCTCTAGAGGAGCCTGTTCTGTAATCGATAAACCCCGA

**SUD112**

>Sud112_clone1 (*Gallus gallus)*

AATCTAGATACCTCCCATCACACATGTATCCGCCTGAGAACTACGAGCACAAACGCTTAAAACTCTAAGGACTTGGCGGTGCCCCAAACCCACCTAGAGGAGCCTGTTCTATAATCGATAA

>Sud112_clone2 (*Gallus gallus*)

AATCTAGATACCTCCCATCACACATGTATCCGCCTGAGAACTACGAGCACAAACGCTTAAAACTCTAAGGACTTGGCGGTGCCCCGAACCCACCTAGAGGAGCCTGTTCTATAATCGATAA

>Sud112_clone3 (*Gallus gallus)*

AATCTAGATACCTCCCATCACACATGTATCCGCCTGAGAACTACGAGCACAAACGCTTAAAACTCTAAGGACTTGGCGGTGCCCCAAACCCACCTAGAGGAGCCTGTTCTATAATCGATAA

**SUD122**

>Sud122_clone1 (*Odocoileus virginianus*)

TAGTTATATAAACAAAACTATTCGCCAGAGTACTACCGGCAATAGCTTAAAACTCAAAGGACTTGGCGGTGCTTTATACCCTTCTAGAGGAGCCTGTTCTATAATCGATAAACCCCGATAGACC

>Sud122_clone2 (*Odocoileus virginianus*)

TAGTTATATAAACAAAACTATTCGCCAGAGTACTACCGGCAATAGCTTAAAACTCAAAGGACTTGGCGGTGCTTTATACCCTTCTAGAGGAGCCTGTTCTATAATCGATAAACCCCGATAGACC

>Sud122_clone3 (*Odocoileus virginianus*)

TAGTTATATAAACAAAACTATTCGCCAGAGTACTACCGGCAATAGCTTAAAACTCAAAGGACTTGGCGGTGCTTTATACCCTTCTAGAGGAGCCTGTTCTATAATCGATAAACCCCGATAGACC

>Sud122_clone4 (*Odocoileus virginianus*)

TAGTTATATAAACAAAACTATTCGCCAGAGTACTACCGGCAATAGCTTAAAACTCAAAGGACTTGGCGGTGCTTTATACCCTTCTAGAGGAGCCTGTTCTATAATCGATAAACCCCGATAGACC

>Sud122_clone5 (*Odocoileus virginianus*)

TAGTTATATAAACAAAACTATTCGCCAGAGTACTACCGGCAATAGCTTAAAACTCAAAGGACTTGGCGGTGCTTTATACCCTTCTAGAGGAGCCTGTTCTATAATCGATAAACCCCGATAGACC

**SUD124**

>Sud124_clone1 (*Zenaida/Columba* sp*.*)

CTAGATGCTTATACTACTAAAGCATCCGCCTGAGGACTACGAGCACAAACGCTTAAAACTCTAAGGACTTGGCGGTGCCCTAAACCCACCTAGAGGAGCCTGTTCTATAATCGATAACCCACGATACACCCGACCACT

>Sud124_clone2 (*Canis lupus*)

CAAAATAATTCGCCAGAGGACTACTAGCAATAGCTTAAAACTCAAAGGACTTGGCGGTGCTTTATATCCCTCTAGAGGAGCCTGTTCTATAATCGATAAACCCCGATAAACCTCGCCACCT

>Sud124_clone3 (*Zenaida/Columba* sp*.*)

CTAGATGCTTATACTACTAAAGCATCCGCCTGAGGACTACGAGCACAAACGCTTAAAACTCTAAGGACTTGGCGGTGCCCTAAACCCACCTAGAGGAGCCTGTTCTATAATCGATAACCCACGATACACCCGACCACT

>Sud124_clone4 (*Zenaida/Columba* sp*.*)

CTAGATGCTTATACTACTAAAGCATCCGCCTGAGGACTACGAGCACAAACGCTTAAAACTCTAAGGACTTGGCGGTGCCCTAAACCCACCTAGAGGAGCCTGTTCTATAATCGATAACCCACGATACACCCGACCACT

**TEY016**

>Tey016_clone1 (*Canis lupus*)

CAAAATAATTCGCCAGAGGACTACTAGCAATAGCTTAAAACTCAAAGGACTTGGCGGTGCTTTATATCCCTCTAGAGGAGCCTGTTCTATAATCGATAAACCCCGATAAAC

>Tey016_clone2 (*Canis lupus*)

CAAAATAATTCGCCAGAGGACTACTAGCAATAGCTTAAAACTCAAAGGACTTGGCGGTGCTTTATATCCCTCTAGAGGAGCCTGTTCTATAATCGATAAACCCCGATAAAC

>Tey016_clone3 (*Canis lupus*)

CAAAATAATTCGCCAGAGGACTACTAGCAATAGCTTAAAACTCAAAGGACTTGGCGGTGCTTTATATCCCTCTAGAGGAGCCTGTTCTATAATCGATAAACCCCGATAAAC

**TEY029**

>Tey029_clone1 (*Canis lupus*)

GATAATTTTACAACAAAATAATTCGCCAGAGGACTACTAGCAATAGCTTAAAACTCAAAGGACTTGGCGGTGCTTTATATCCCTCTAGAGGAGCCTGTTCTATAATCGATAAACCCCGATAAACCTCACCAC

>Tey029_clone2 (*Canis lupus*)

GATAATTTTACAACAAAATAATTCGCCAGAGGACTACTAGCAATAGCTTAAAACTCAAAGGACTTGGCGGTGCTTTATATCCCTCTAGAGGAGCCTGTTCTATAATCGATAAACCCCGATAAACCTCACCAC

>Tey029_clone3 (*Canis lupus*)

GATAATTTTACAACAAAATAATTCGCCAGAGGACTACTAGCAATAGCTTAAAACTCAAAGGACTTGGCGGTGCTTTATATCCCTCTAGAGGAGCCTGTTCTATAATCGATAAACCCCGATAAACCTCACCAC

>Tey029_clone4 (*Canis lupus*)

GATAATTTTACAACAAAATAATTCGCCAGAGGACTACTAGCAATAGCTTAAAACTCAAAGGACTTGGCGGTGCTTTATATCCCTCTAGAGGAGCCTGTTCTATAATCGATAAACCCCGATAAACCTCACCAC

>Tey029_clone5 (*Canis lupus*)

GATAATTTTACAACAAAATAATTCGCCAGAGGACTACTAGCAATAGCTTAAAACTCAAAGGACTTGGCGGTGCTTTATATCCCTCTAGAGGAGCCTGTTCTATAATCGATAAACCCCGATAAACCTCACCAC

>Tey029_clone6 (*Canis lupus*)

GATAATTTTACAACAAAATAATTCGCCAGAGGACTACTAGCAATAGCTTAAAACTCAAAGGACTTGGCGGTGCTTTATATCCCTCTAGAGGAGCCTGTTCTATAATCGATAAACCCCGATAAACCTCACCAC

>Tey029_clone7 (*Canis lupus*)

GATAATTTTACAACAAAATAATTCGCCAGAGGACTACTAGCAATAGCTTAAAACTCAAAGGACTTGGCGGTGCTTTATATCCCTCTAGAGGAGCCTGTTCTATAATCGATAAACCCCGATAAACCTCACCAC

>Tey029_clone8 (*Canis lupus*)

GATAATTTTACAACAAAATAATTCGCCAGAGGACTACTAGCAATAGCTTAAAACTCAAAGGACTTGGCGGTGCTTTATATCCCTCTAGAGGAGCCTGTTCTATAATCGATAAACCCCGATAAACCTCACCAC

**TEY033**

>Tey033_ clone1 (*Sus scrofa*)

CCAAATAGTTACATAACAAAACTATTCGCCAGAGTACTACTCGCAACTGCCTAAAACTCAAAGGACTTGGCGGTGCTTCACATCCACCTAGAGGAGCCTGTTCTATAATCGATAAACCCCGATAGACCTTACCA

>Tey033_ clone2 (*Homo sapiens*)

ACAGTTAAATCAACAAAACTGCTCGCCAGAACACTACGAGCCACAGCTTAAAACTCAAAGGACCTGGCGGTGCTTCATATCCCTCTAGAGGAGCCTGTTCTGTAATCGATAAACCCCGATCAACCTCACCAC

**TEY036**

>Tey036_clone1 (*Canis lupus*)

AACATAGATAATTTTACAACAAAATAATTCGCCAGAGGACTACTAGCAATAGCTTAAAACTCAAAGGACTTGGCGGTGCTTTATATCCCTCTAGAGGAGCCTGTTCTATAATCGATAAACCCCGATAAACCTCACCACCTTTCGC

>Tey036_clone2 (*Homo sapiens*)

TTAAATCAACAAAACTGCTCGCCAGAACACTACGAGCCACAGCTTAAAACTCAAGGGACCTGGCGGTGCTTCATATCCCTCTAGAGGAGCCTGTTCTGTAATCGATAAACCCCGATCAACCTCACCACCTC

>Tey036_clone3 (*Homo sapiens*)

TTAAATCAACAAAACTGCTCGCCAGAACACTACGAGCCACAGCTTAAAACTCAAAGGACCTGGCGGTGCTTCATATCCCTCTAGAGGAGCCTGTTCTGTAATCGATAAACCCCGATCAACCTCACCACCTC

>Tey036_clone4 (*Homo sapiens*)

TTAAATCAACAAAACTGCTCGCCAGAACACTACGAGCCACAGCTTAAAACTCAAAGGACCTGGCGGTGCTTCATATCCCTCTAGAGGAGCCTGTTCTGTAATCGATAAACCCCGATCAACCTCACCACCTC

>Tey036_clone5 (*Homo sapiens*)

TTAAATCAACAAAACTGCTCGCCAGAACACTACGAGCCACAGCTTAAAACTCAAAGGACCTGGCGGTGCTTCATATCCCTCTAGAGGAGCCTGTTCTGTAATCGATAAACCCCGATCAACCTCACCACCTC

**TEY043**

>Tey043_clone1 (*Zenaida/Columba* sp*.*)

AATCTAGATGCTTATACTACTAAAGCATCCGCCTGAGGACTACGAGCACAAACGCTTAAAACTCTAAGGACTTGGCGGTGCCCTAAACCCACCTAGAGGAGCCTGTTCTATAATCGATAACCCACGATACACCCGACCA

**TEY049**

>Tey049_clone1 (*Homo sapiens*)

CAGAACACTACGAGCCACAGCTTAAAACTCAAAGGACCTGGCGGTGCTTCATATCCCTCTAGAGGAGCCTGTTC

>Tey049_clone2 (*Canis lupus*)

ACATAGATAATTTTACAACAAAATAATTCGCCAGAGGACTACTAGCAATAGCTTAAAACTCAAAGGACTTGGCGGTGCTTTATATCCCTCTAGAGGAGCCTGTTCTATAATCGACAAACCCCGATAAACCTCACCAC

>Tey049_clone3 (*Homo sapiens*)

CAGAACACTACGAGCCACAGCTTAAAACTCAAAGGACCTGGCGGTGCTTCATATCCCTCTAGAGGAGCCTGTTC

**TEY060**

>Tey060_clone1 (*Bos* sp.) *

CAGATAATTACATAAACAAAATTATTCGCCAGAGTACTACTAGCAACAGCTTAAAACTCAAAGGACTTGGCGGTGCTTTATATCCTTCTAGAGGAGCCTGTTCTATAATCGATAAACCCCGATAAACCTCACCAATTCTTGCTAATACAGTCTATATACCGCCATCTTCAGCAAACA

**TEY066**

>Tey066_clone1 (*Bos* sp*.*)

GCAACAGCTTAAAACTCAAAGGACTTGGCGGTGCTTTATATCCTTCTAGAGGAGCCTGTTCTATAATCGATAAACCCCGATAAACCTCACCAAT

>Tey066_clone2 (*Bos* sp*.*)

ACAGATAATTACATAAACAAAATTATTCGCCAGAGTACTACTAGCAACAGCTTAAAACTCAAAGGACTTGGCGGTGCTTTATATCCTTCTAGAGGAGCCTGTTCTATAATCGATAAACCCCGATAAACCTCACCAAT

**TEY071**

>Tey071_clone1 (*Bos* sp*.*) *

AGCAACAGCTTAAAACTCAAAGGACTTGGCGGTGCTTTATATCCTTCTAGAGGAGCCTGTTCTATAATCGATAAACCCCGATAAACCTCACCAATTCTTGCTAATACAGTCTATATACCGCCATCTTCAGCAAACA

>Tey071_clone2 (*Bos* sp*.*) *

AGCAACAGCTTAAAACTCAAAGGACTTGGCGGTGCTTTATATCCTTCTAGAGGAGCCTGTTCTATAATCGATAAACCCCGATAAACCTCACCAATTCTTGCTAATACAGTCTATATACCGCCATCTTCAGCAAACA

>Tey071_clone3 (*Bos* sp*.*) *

AGCAACAGCTTAAAACTCAAAGGACTTGGGGGTGCTTTATATCCTTCTAGAGGAGCCTGTTCTATAATCGATAAACCCCGATAAACCTCACCAATTCTTGCTAATACAGTCTATATACCGCCATCTTCAGCAAACA

>Tey071_clone4 (*Bos* sp*.*) *

AGCAACAGCTTAAAACTCAAAGGACTTGGCGGTGCTTTATATCCTTCTAGAGGAGCCTGTTCTATAATCGATAAACCCCGATAAACCTCACCAATTCTTGCTAATACAGTCTATATACCGCCATCTTCAGCAAACA

>Tey071_clone5 (*Bos* sp.) *

AGCAACAGCTTAAAACTCAAAGGACTTGGCGGTGCTTTATATCCTTCTAGAGGAGCCTGTTCTATAATCGATAAACCCCGATAAACCTCACCAATTCTTGCTAATACAGTCTATATACCGCCATCTTCAGCAAACA

>Tey071_clone6 (*Bos* sp.) *

AACTCAAAGGACTTGGCGGTGCTTTATATCCTTCTAGAGGAGCCTGTTCTATAATCGATAAACCCCGATAAACCTCACCAATTCTTGCTAATACAGTCTATATACCGCCATCTTCAGCAAACA

**TEY073**

>Tey073_clone1 (*Homo sapiens*)

ACAAAACTGCTCGCCAGAACACTACGAGCCACAGCTTAAAACTCAAAGGACCTGGCGGTGCTTCATATCCCTCTAGAGGAGCCTGTTCTGTAATCGATAAACCCCGATCAACCTCAC

>Tey073_clone2 (*Homo sapiens*)

ACAAAACTGCTCGCCAGAACACTACGAGCCACAGCTTAAAACTCAAAGGACCTGGCGGTGCTTCATATCCCTCTAGAGGAGCCTGTTCTGTAATCGATAAACCCCGATCAACCTCAC

>Tey073_clone3 (*Homo sapiens*) **

AAAAAACTGTTCGCCAGAACACTACAAGCAACAGCTTAAAACTCAAAGGACTTGGCGGTGCTTTATATCCCTCTAAAGGAGCCTGTTCTGTAATCGATAAACCCCGATCAACCTCAA

**TEY074**

>Tey074_clone1 (*Bos* sp.)

ACAGATAATTACATAAACAAAATTATTCGCCAGAGTACTACTAGCAACAGCTTAAAACTCAAAGGACTTGGCGGTGCTTTATATCCTTCTAGAGGAGCCTGTTCTATAATCGATAAACCCCGATAAACCTCACCAAT

>Tey074_clone2 (*Homo sapiens*) *^,^ **

ACTACAAGCAACAGCTTAAAACTCAAAGGACTTGGCGGTGCTTTATATCCCTCTAAAGGAACCTGTTCTATAATCGATAAACCCCAATTTACCTCACCACCGC

**TEY075**

>Tey075_clone1 (*Homo sapiens*)

AATCAACAAAACTGCTCGCCAGAACACTACGAGCCACAGCTTAAAACTCAAAGGACCTGGCGGTGCTTCATATCCCTCTAGAGGAGCCTGTTCTGTAATCGAT

>Tey075_clone2 (*Homo sapiens*)

AATCAACAAAACTGCTCGCCAGAACACTACGAGCCACAGCTTAAAACTCAAAGGGCCTGGCGGTGCTTCATATCCCTCTAGAGGAGCCTGTTCTGTAATCGAT

>Tey075_clone3 (*Homo sapiens*)

AATCAACAAAACTGCTCGCCAGAACACTACGAGCCACAGCTTAAAACTCAAAGGACCTGGCGGTGCTTCATATCCCTCTAGAGGAGCCTGTTCTGTAATCGAT

>Tey075_clone4 (*Homo sapiens*)

AATCAACAAAACTGCTCGCCAGAACACTACGAGCCACAGCTTAAAACTCAAAGGACCTGGCGGTGCTTCATATCCCTCTAGAGGAGCCTGTTCTGTAATCGAT

**TEY077**

>Tey077_clone1 (*Homo sapiens*) *

CCACAGCTTAAAACTCAAAGGACCTGGCGGTGCTTCATATCCCTCTAGAGGAGCCTGTTCTGTAATCGATAAACCCCGATCAACCTCACCACCTCTTGCTCAGCCTATATACCGCCATCTTCAGCAAACA

**TEY139**

>Tey139_clone1 (*Homo sapiens*)

GTTAAATCAACAAAACTGCTCGCCAGAACACTACGAGCCACAGCTTAGAACTCAAAGGACCTGGCGGTGCTTCATATCCCTCTAGAGGAGCCTGTTCTGTAATCGATAAACCCCGA

>Tey139_clone2 (*Homo sapiens*)

CTCAACAGTTAAATCAACAAAACTGCTCGCCAGAACACTACGAGCCACAGCTTAAAACTCAAAGGACCTGGCGGTGCTTCATATCCCTCTAGAGGAGCCTGTTCTGTAATCG

>Tey139_clone 3 (*Homo sapiens*)

ACAGTTAAATCAACAAAACTGCTCGCCAGAACACTACGAGCCACAGCTTAAAACTCAAAGGACCTGGCGGTGCTTCATATCCCTCTAGAGGAGCCTGTTCTGTAATCGATAAACCCCGA

>Tey139_clone 4 (*Homo sapiens*)

CTAAACCTCAACAGTTAAATCAACAAAACTGCTCGCCAGAACACTACGAGCCACAGCTTAAAACTCAAAGGACCTGGCGGTGCTTCATATCCCTCTAGAGGAGCCTGTTCTGTAATCGATAAACCCCGATCAACCTCAC

>Tey139clone 5 (*Homo sapiens*)

CAGTTAAATCAACAAAACTGCTCGCCAGAACACTACGAGCCACAGCTTAAAACTCAAAGGACCTGGCGGTGCTTCATATCCCTCTAGAGGAGCCTGTTCTGTAATCGATAAACCCCGA

**Control 1**

>Control 1_clone1 (*Columba livia*)

GCATCCGCCTGAGAACTACGAGCACAAACGCTTAAAACTCTAAGGACTTGGCGGTGCCCCAAACCCACCTAGAGGAGCCTGTTCTGTAATCGATACTCCACGATACAC

>Control 1_clone2 (*Columba livia*)

TATATAACCAAAGCATCCGCCTGAGAACTACGAGCACAAACGCTTAAAACTCTAAGGACTTGGCGGTGCCCCAAACCCACCTAGAGGAG

**Control 2**

>Control 2_clone1 (*Columba livia)* *

CCAAACTGGGATTAGATACCCCACTATGCCTGGCCCTAAATCTTGATGCTCTATACAACCAAAGCATCCGCCTGAGAACTACGAGCACAAACGCTTAAAACTCTAAGGACTTGGCGGTGCCCCAAACCCACATAGAGGA

>Control 2_clone2 (*Columba livia*) *

CCAAACTGGGATTAGATACCCCACTATGCCTGGCCCTAAATCTTGATGCTCTATACAACCAAAGCATCCGCCTGAGAACTACGAGCACAAACGCTTAAAACTCTAAGGACTTGGCGGTGCCCCAAACCCACATAGAGGA

>Control 2_clone3 (*Columba livia*) *

CCAAACTGGGATTAGATACCCCACTATGCCTGGCCCTAAATCTTGATGCTCTATACAACCAAAGCATCCGCCTGAGAACTACGAGCACAAACGCTTAAAACTCTAAGGACTTGGCGGTGCCCCAAACCCACATAGAGGA

>Control 2_clone4 (*Columba livia*) *

CCAAACTGGGATTAGATACCCCACTATGCCTGGCCCTAAATCTTGATGCTCTATACAACCAAAGCATCCGCCTGAGAACTACGAGCACAAACGCTTAAAACTCTAAGGACTTGGCGGTGCCCCAAACCCACATAGAGGA

>Control 2_clone5 (*Columba livia*) *

CCAAACTGGGATTAGATACCCCACTATGCCTGGCCCTAAATCTTGATGCTCTATACAACCAAAGCATCCGCCTGAGAACTACGAGCACAAACGCTTAAAACTCTAAGGACTTGGCGGTGCCCCAAACCCACATAGAGGA

>Control 2_clone6 (*Columba livia*) *

CCAAACTGGGATTAGATACCCCACTATGCCTGGCCCTAAATCTTGATGCTCTATACAACCAAAGCATCCGCCTGAGAACTACGAGCACAAACGCTTAAAACTCTAAGGACTTGGCGGTGCCCCAAACCCACATAGAGGA

>Control 2_clone7 (*Columba livia*) *

CCAAACTGGGATTAGATACCCCACTATGCCTGGCCCTAAATCTTGATGCTCTATACAACCAAAGCATCCGCCTGAGAACTACGAGCACAAACGCTTAAAACTCTAAGGACTTGGCGGTGCCCCAAACCCACATAGAGGA

>Control 2_clone8 (*Homo sapiens*)

ACAGTTAAATCAACAAAACTGCTCGCCAGAACACTACGAGCCACAGCTTAAAACTCAAAGGACCTGGCGGTGCTTCATATCCCTCTAGAGGAGCCTGTTCTGTAATCGATAAACCCCGATCAACCTCACCACCTC

**Control 3**

>Control 3_clone1 (*Streptopelia decaocto)*

ATCTTAATGCTCTATACAACCAAAGCATCCACCTGAGAACTACGAGCACGAACGCTTAAAACTCTAAGGACTTGGCGGTGCCCCAAACCCACATAGAGGAGCCTGTTCTATAATCGATAACCCAAGATACACCTGACCACT

>Control 3_clone2 (*Streptopelia/Columba* sp*.)*

AATCTTGATGCTCTATACAACCAAAGCATCCACCTGAGAACTACGTGCACAAACGCTTAAAACTCTAAGGACATGGCGGTGCCCCAAACCCACCTAGAGGAGCCTGTTCTATAATCGATAACCCAAGATACACCTGACCACT

>Control 3_clone3 (*Streptopelia decaocto)*

GCTCTATACAACCAAAGCATCCACCTGAGAACTACGAGCACAAATGCTTAAAACTCTAAGGACTTGGCGGTGCCCCAAACCCACCTAGAGGAGCCTGTTCTATAATTGATAACCCACGATACAC

>Control 3_clone4 (*Streptopelia decaocto)*

GATGCTCTATACAACCAAAGCATCCGCCTGAGAACTACGAGCACAAACGCTTAAAACTCTAAGGACTTGGCGGTGCCCCAAACCCACMTAGAGGAGCCTGTTCTATAATCGATAACCCA

>Control 3_clone5 (*Streptopelia decaocto)*

TCTTGATGCTCTATACAACCAAAGCATCCRCCTGAGAACTACGAGCACAAACGCTTAAAACTCTAAGGACTTGGCGGTGCCCCAAACCCACMTAGAGGAGCCTGTTCTATAATCGATAACCCA

>Control 3_clone6 (*Streptopelia decaocto)*

TCTTGATGCTCTATACAACCAAAGCATCCGCCTGAGAACTACGAGCACAAACGCTTAAAACTCTAAGGACTTGGCGGTGCCCCAAACCCACATAGAGGAGCCTGTTCTATAATCGATAACCCAAGATACACC

* These sequences were recovered from only one strand.

** These divergent human sequences presented highest identity with sequences of human nuclear DNA, and likely correspond to nuclear insertions of mitochondrial origin (NumtS) which were amplified with our primers, as reviewed for example in [70].

References:

[70] Dayama G, Emery SB, Kidd JM, Mills RE. The genomic landscape of polymorphic human nuclear mitochondrial insertions. Nucleic Acids Res. 2014;42(20):12640-9.
