# Supplementary material for: Disentangling Trypanosoma cruzi transmission cycle dynamics through the identification of blood meal sources of natural populations of Triatoma dimidiata in Yucatán, Mexico
Source: Parasit Vectors. 2019 Nov 29;12:572. doi: 10.1186/s13071-019-3819-7 (PMC6884771; doi:10.1186/s13071-019-3819-7)

**Additional file 3: Figure S1. Rarefaction curves of *Triatoma dimidiata* blood meal source diversity.** Blood meal sources were plotted against the number of partial *12S* rRNA sequences analyzed to assess coverage of the diversity of blood meal sources of *T. dimidiata*. For blood meal sources identified from bugs collected within the villages (blue line), the plateau was almost reached, suggesting that most of the diversity of blood meal sources in this environment were identified in our sampling. For blood meal sources identified from bugs collected within sylvatic ecotopes (red line), the observed exponential phase suggests that the diversity of blood meal sources of *T. dimidiata* in this environment was not totally identified.


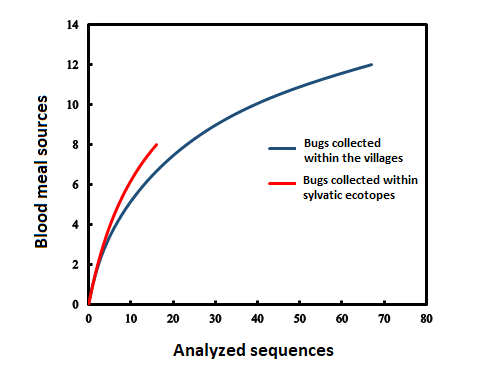

Supplement: Supplementary file 3 — Additional file 3: Figure S1. Rarefaction curves of Triatoma dimidiata blood meal source diversity. [file 13071_2019_3819_MOESM3_ESM.docx]
